# TSLP links intestinal nutrient sensing with amplification of the ILC2–tuft cell circuit

**DOI:** 10.1038/s41590-025-02328-y

**Published:** 2025-11-12

**Authors:** Chang Liao, Elvira Mennillo, Michael G. Kattah, Ming Ji, Zhi-En Wang, Daniel J. Drucker, Hong-Erh Liang, Richard M. Locksley

**Affiliations:** 1 Department of Medicine, University of California, San Francisco, CA USA; 2 Howard Hughes Medical Institute, University of California, San Francisco, CA USA; 3 Lunenfeld-Tanenbaum Research Institute, Mount Sinai Hospital, Toronto, Ontario Canada

**Keywords:** Mucosal immunology, Innate lymphoid cells

## Abstract

Group 2 innate lymphocytes (ILC2s) are prevalent in small intestine but their role during homeostasis is unclear. Here we show that thymic stromal lymphopoietin (TSLP)—a cytokine implicated in ILC2 activation—is expressed constitutively in subepithelial fibroblasts, including telocytes and crypt-associated trophocytes, which are specialized fibroblasts necessary to sustain epithelial identity. Feeding increases TSLP and induces ILC2 type 2 cytokines that are attenuated by deletion of TSLP in fibroblasts or TSLP receptor on ILC2s. Both mouse and human intestinal fibroblasts express receptors for glucagon-like peptide-2 (GLP-2)—an intestinotrophic growth factor released by enteroendocrine cells following food intake. GLP-2 promotes intestinal TSLP in mouse and human intestinal fibroblasts, and TSLP-dependent ILC2 activation and tuft cell hyperplasia in mice, thus linking nutrient detection with ILC2-mediated amplification of the tuft cell chemosensory circuit that promotes epithelial surveillance of ingested cargo.

## Abstract (web summary)

Locksley and colleagues describe a nutrient-sensing circuit in the small intestine. Upon feeding, TSLP production from fibroblasts is increased in a GLP-2-dependent manner, resulting in increased ILC2 activation and tuft cell hyperplasia, thus linking food intake with ILC2 activation.

## Main

The small intestinal mucosa represents a key barrier balancing host nutritional needs and protection from potentially noxious chemical and infectious environmental agents. Innate and innate-like lymphocytes, including group 3 innate lymphoid cells, intraepithelial lymphocytes, γẟ T cells, specialized RORγt^+^ antigen-presenting cells and regulatory T cells, create a healthy microenvironment that sustains the dynamic interface facilitating nutrient absorption, metabolism, microbiota control, tolerance and epithelial protection during fasting, feeding and circadian cycles^1–5^. ILC2s are prevalent in small intestinal lamina propria (siLP) although their participation in resting homeostasis is unclear. ILC2s and adaptive Th2 cells become highly engaged during helminth infection and in food allergy but neither of these conditions necessarily explains the basal residence of innate ILC2s in small intestine siLP.

In previous studies, our laboratory and others identified a key role for the alarmin interleukin (IL)-25 as a tuft cell-derived cytokine that activates siLP ILC2s to release IL-13, which biases epithelial progenitors to a secretory cell fate that increases goblet and tuft cells in a forward-amplifying circuit accounting for the ‘weep and sweep’ response to helminths and protists^6–8^. Deletion of cell intrinsic regulators of IL-25 signaling, such as A20 (encoded by the *Tnfaip3* gene), resulted in constitutive activation of the circuit, suggesting control of the basal state^9^. The combinatorial role of alarmins in regulating the activation of tissue resident ILC2s and Th2 cells^10,11^ prompted us to consider roles for additional alarmins in small intestinal physiology and homeostasis. Here we demonstrate that TSLP produced by subepithelial fibroblasts relays signals from epithelial enteroendocrine cells (EECs) to siLP ILC2s to amplify the tuft cell circuit by a reversible process responsive to food intake.

## Specialized subepithelial fibroblasts express TSLP in the small intestine

**Fig. 1:**
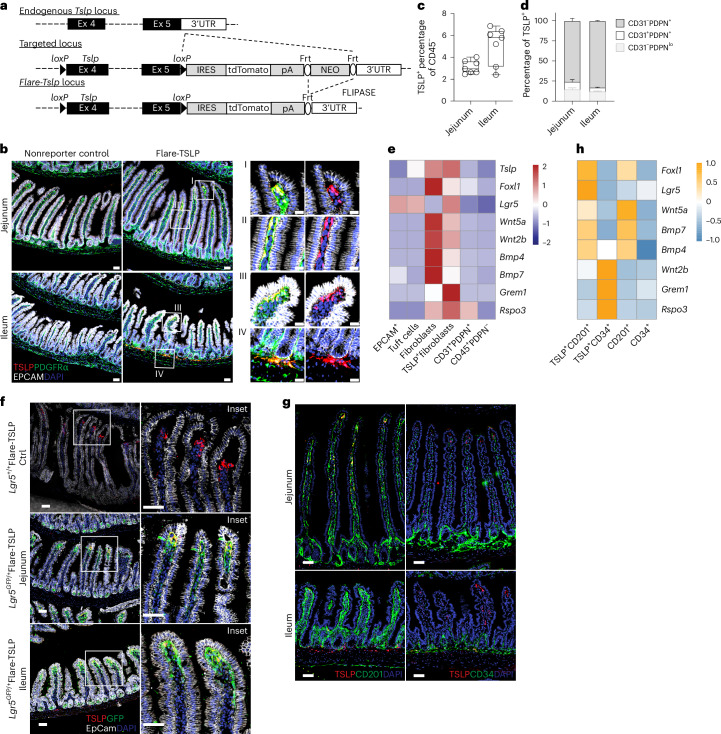
Stromal cells are primary sources of TSLP in the small intestine. **a**, Gene targeting strategy for the flox-and-reporter of *Tslp* locus. Frt, target site for FLIPASE recombinase; pA, bovine growth hormone poly(A) tail; UTR, untranslated region. **b**, Representative imaging of jejunum (top) and ileum (bottom) of Flare-TSLP or nonreporter control mice. Red, TSLP-tdTomato; green, PDGFRα; white, EPCAM; blue, DAPI; ×20 objective. Scale bars, 50 µm (main images) and 20 µm (boxed areas I–IV). **c**,**d**, Flow cytometric analysis of stromal populations in jejunum and ileum siLP in Flare-TSLP mice. **c**, Percentage of total TSLP-tdTomato^+^ cells in CD45^−^ population; *N* = 7 biological replicates each column group. **d**, Percentage of endothelial and nonendothelial stromal cells within TSLP-tdTomato^+^ cells. Cells were stained with anti-CD31 and anti-PDPN antibodies; *N* = 3 biological replicates each column group. Error bars indicate samples mean ± s.e.m. **e**, RT–qPCR analysis of sorted CD45^−^EPCAM^+^ epithelial cells, CD45^−^EPCAM^+^ SiglecF^+^ tuft cells, CD45^−^CD31^−^PDPN^+^ fibroblasts, CD45^−^CD31^−^PDPN^+^TSLP-tdTomato^+^fibroblasts, and CD45^−^CD31^+^PDPN^+^ lymphatic endothelial and CD45^+^PDPN^−^ hematopoietic cells from small intestinal epithelial fraction or siLP (whole tissue) of Flare-TSLP mice. Gene expression normalized to 18S. **f**, Representative imaging of jejunum (middle) and ileum (bottom) of Flare-TSLP; *Lgr5*-eGFP dual reporter mice or Flare-TSLP single reporter (Ctrl) mice (top). Note that epithelial tuft cells can express *Lgr5*-eGFP. Red, TSLP-tdTomato; green, *Lgr5*-eGFP; white, EPCAM; blue, DAPI; ×20 objective. Scale bars, 50 µm. **g**, Representative imaging of CD201 and CD34 expression in jejunum (top) and ileum (bottom) of Flare-TSLP mice. Red, TSLP-tdTomato; green, CD201 (left) or CD34 (right); blue, DAPI; ×20 objective. Scale bars, 100 µm. **h**, RT–qPCR analysis of sorted CD45^−^CD31^−^ TSLP-tdTomato^+^CD201^+^CD31^−^, CD45^−^CD31^−^TSLP-tdTomato^+^CD34^+^, CD45^−^CD31^−^CD201^+^ and CD45^−^CD31^−^CD34^+^ stromal subpopulations from siLP (whole tissue) of Flare-TSLP mice. Gene expression normalized to 18S.

**Extended Data Fig. 1:**
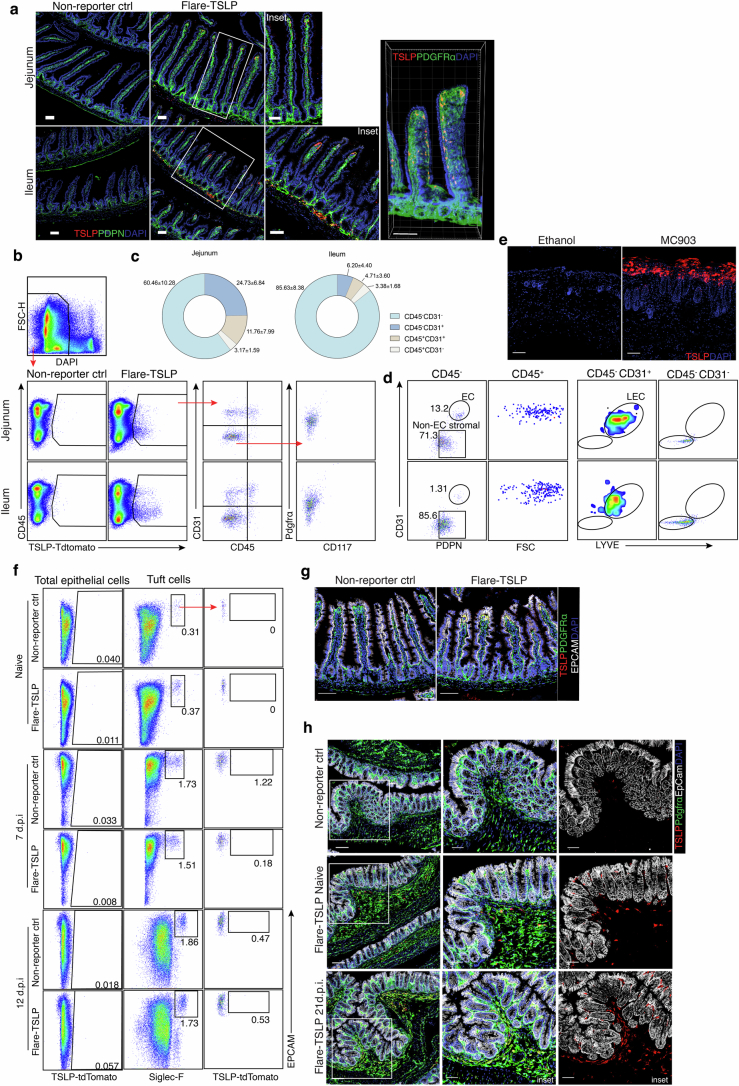
Characterization of Flare-TSLP reporter mice. **a**, Representative imaging showing jejunum (**top**) and ileum (**bottom**) of Flare-TSLP mice or non-reporter control mice. Red, TSLP-tdTomato; Green, PDPN; Blue, DAPI. 20x objective. Scale bar, 50 µm. **Right panel**, max intensity projection of three - dimensional imaging showing jejunual tissues from Flare-TSLP mice; scale bar, 100 µm. **b-d**, Flow cytometric analysis of CD45^+^ and stromal populations in jejunal and ileal lamina propria in Flare-TSLP or non-reporter control (ctrl) mice. **b**, CD45^+^ cells were CD31^+^ endothelial cells. CD45^−^CD31^−^ stromal cells were PDGFRa^+^cKit^−^ (non-Cajal cells, which are cKit^+^). **c**, Proportions of CD45^+^CD31^+^, CD45^+^CD31^−^, CD45^−^CD31^+^, and CD45^−^CD31^−^ cells in TSLP-tdTomato^+^ cells represented by pie charts. **d**, TSLP-tdTomato^+^CD45^−^CD31^+^ cells were PDPN^+^ and LYVE1^+^ consistent with lymphatic endothelial cells (LECs). TSLP-tdTomato^+^CD45^+^ cells are CD31^+^ FSC^hi^ large cells consistent with a non-hematopoietic lineage. **e**, Representative imaging showing back skin of Flare-TSLP mice treated with ethanol or calcipotriol (MC903) for 7 days. Red, TSLP-tdTomato; Blue, DAPI. 20x objective. Scale bar, 100 µm. **f**, Flow cytometric analysis of epithelial TSLP expression in proximal small intestine in naive or on day 7 and 12 post *N. brasiliensis* infection in Flare-TSLP or non-reporter mice. d.p.i., day post infection. **g-h**, Representative imaging of jejunum in Flare-TSLP or non-reporter mice on day 5 after *N. brasiliensis* (**g**) and in cecum day 21 after *T. muris* infection (**h**). Red, TSLP-tdTomato; Green, PDGFRa; White, EPCAM; Blue, DAPI. 20x objective. Scale bar, 100 µm. Inset scale bar, 50 µm. All data are biological replicates, n≥3. Data are representative of at least two independent experiments.

We targeted the *Tslp* locus to identify TSLP-producing cells using the flox-and-reporter (Flare) cassette employed previously to visualize IL-25-producing tuft cells^6^ (Fig. 1a). Under basal conditions in specific pathogen-free C57BL/6 Flare-TSLP mice, the predominant TSLP reporter-positive (TSLP-tandem-dimer red fluorescent protein (tdTomato)^+^) cells in the small intestine were subepithelial PDGFRα^+^Podoplanin (PDPN)^+^ cells with the morphology of fibroblasts along the villi and enriched at the villus tips throughout small intestine and among crypt-associated cellular networks in the jejunum and ileum (Fig. 1b, Extended Data Fig. 1a and Supplementary Videos 1–7). These cells comprised >80% of TSLP-tdTomato^+^ cells among CD45^−^ cells, with their prevalence increasing from 2–4% in the proximal small intestine to 2–7% in the distal small intestine (Fig. 1c). TSLP-tdTomato^+^CD31^+^ cells were PDPN^+^ LYVE1^+^ lymphatic endothelial cells (LECs) and were less abundant, comprising 10–20% and 1–10% of TSLP-tdTomato^+^CD45^−^ cells in the proximal and distal small intestine, respectively (Fig. 1d and Extended Data Fig. 1b–d). TSLP-tdTomato^+^CD45^+^ cells were rare and found only among the CD31^+^ endothelial cells (Extended Data Fig. 1d). Despite reports of TSLP-positive small intestinal epithelia^12,13^, including type 2 tuft cells, we did not identify intestinal TSLP-tdTomato^+^ epithelial cells by flow cytometry or microscopy in Flare-TSLP mice (Fig. 1b and Extended Data Fig. 1f). This was not due to an inability to visualize epithelial TSLP, as topical application of the vitamin D analog calcipotriol (MC903), known to induce TSLP in keratinocytes^14^, induced reporter signal in Flare-TSLP mice (Extended Data Fig. 1e). In the small intestine, epithelial tuft cells remained TSLP reporter-negative throughout a kinetic analysis of acute *Nippostrongylus brasiliensis* helminth infection (Extended Data Fig. 1f,g). Similarly, proximal large intestine epithelial cells remained TSLP reporter-negative after *Trichuris muris* helminth infection, which is known to require TSLP^12^ (Extended Data Fig. 1h). After both infections, however, reporter expression was present in PDGFRα^+^ subepithelial fibroblasts (Extended Data Fig. 1g,h).

**Extended Data Fig. 2:**
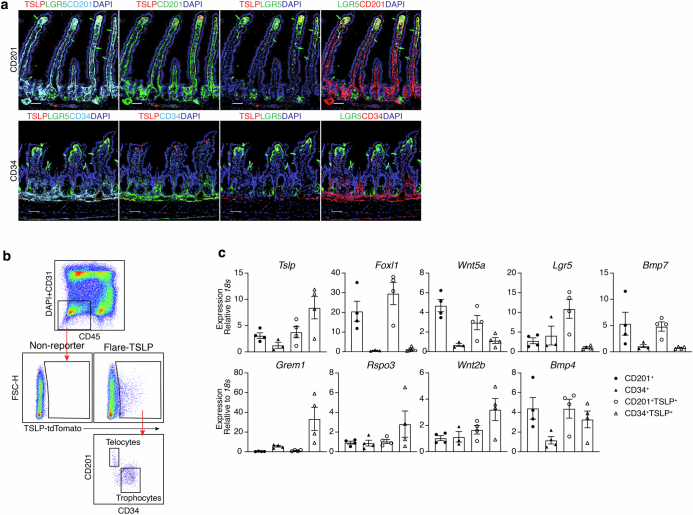
Analysis of CD201^+^ telocytes and CD34^+^ trophocytes. **a**, Representative imaging of CD201 and CD34 expression in proximal and distal small intestine of Flare-TSLP; *Lgr5*-eGFP dual reporter mice. Note that epithelial tuft cells can express *Lgr5*-eGFP. Scale bar, 100 µm. **b**, Gating strategy for FACS sorting of CD45^−^CD31^−^TSLP-tdTomato^+^CD201^+^, and CD45^−^CD31^−^TSLP-tdTomato^+^CD34^+^ cells from small intestinal LP (siLP, whole tissue) of Flare-TSLP mice. **c**, RT-qPCR analysis for *Tslp, Foxl1, Wnt5a, Lgr5, Bmp7, Grem1, Rspo3, Wnt2b, Bmp4* expression in sorted CD45^−^CD31^−^TSLP-tdTomato^+^CD201^+^, and CD45^−^CD31^−^TSLP-tdTomato^+^CD34^+^, CD45^−^CD31^−^CD201^+^, and CD45^−^CD31^−^CD34^+^ stromal cells from siLP (whole tissue) of Flare-TSLP mice. Data are biological replicates, n≥3. Data are representative of at least two independent experiments. Error bars indicate samples mean ± SEM.

The location and morphology of constitutive TSLP-tdTomato^+^ subepithelial fibroblasts in Flare-TSLP mice resembled previous reports of telocytes and crypt-associated trophocytes. These structural cells generate the counter-regulating WNT and BMP gradients that maintain the crypt stem cell niche and regulate epithelial differentiation during vertical zonation of the villi^15,16^. To confirm the identity of these cells, we employed fluorescence-activated cell sorting (FACS) to enrich TSLP-tdTomato^+^ cells and used quantitative PCR (qPCR) with reverse transcription (RT) (RT–qPCR) to assess the expression of signature transcripts for telocytes, including *Foxl1*, *Wnt5a* and *Bmp7*, and trophocytes, including *Wnt2b*, *Grem1* and *Rspo3* (refs. ^15,16^; Fig. 1e). Expression of these genes was relatively low in epithelial cell adhesion molecule (EPCAM)^+^ total epithelial cells, tuft cells, CD45^−^CD31^+^PDPN^+^ LECs or CD45^+^PDPN^−^ hematopoietic cells, with the exception of *Rspo3*, which was also highly expressed by LECs (Fig. 1e). The expression of *Lgr5* in the TSLP-tdTomato^+^ population (Fig. 1e) suggested that villus tip telocytes (VTTs), specialized telocytes that act as signaling hubs to direct apical epithelial cell fate^17^, also express TSLP. Using Flare-TSLP;*Lgr5*-eGFP dual reporter mice, we confirmed co-expression of *Tslp* and *Lgr5* among VTTs (Fig. 1f). Previous studies identified CD201 and CD34 as markers for intestinal telocytes and trophocytes, respectively^16,18^. In line with this, CD201 labeled villus subepthelial fibroblasts and *Lgr5*-GFP^+^ VTTs but not trophocytes, while CD34 labeled crypt-associated TSLP-tdTomato^+^ trophocytes (Fig. 1g and Extended Data Fig. 2a). We sorted CD45^−^CD31^−^CD201^+^TSLP-tdTomato^+^ and CD45^−^CD31^−^CD34^+^TSLP-tdTomato^+^ cells (Extended Data Fig. 2b) and confirmed their expression of telocyte- and trophocyte-associated transcripts, respectively (Fig. 1h and Extended Data Fig. 2c). Thus, distinct subepithelial fibroblasts, including villus telocytes and trophocytes, as well as LECs, constitute the primary cell types that express TSLP in the mouse small intestine under resting conditions.

## TSLP increases with feeding and activates ILC2 cytokine production

**Fig. 2:**
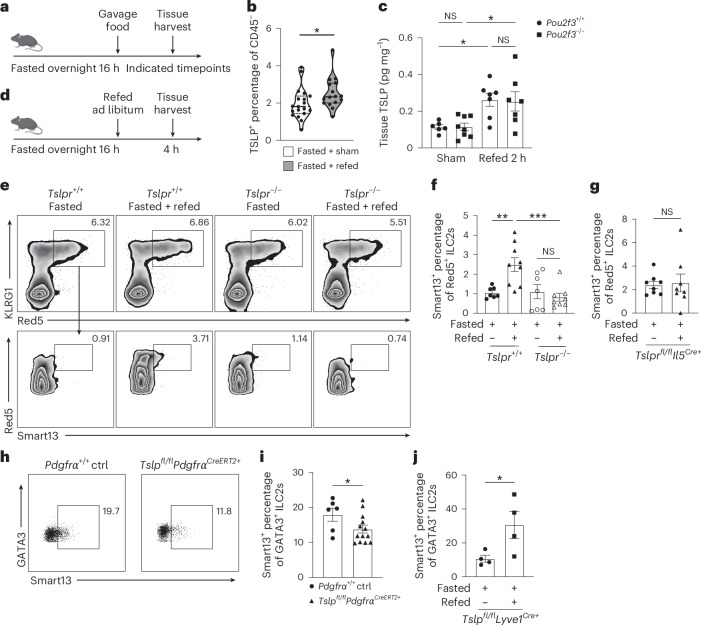
Feeding increases intestinal TSLP and drives ILC2 activation. **a**, Schematic of the protocol for measuring intestinal TSLP after feeding. Mice were fasted for 16 h overnight before oral gavage with 500 μl food slurry (refed) or water as volumetric control (sham). Tissues were harvested as indicated. **b**, Percentage of TSLP-tdTomato^+^ cells among CD45^−^ cells in proximal jejunal siLP by flow cytometric analysis after oral gavage at 2 h after overnight fasting. Biological replicates *N* = 19 for sham control group and *N* = 16 for refeeding group. Statistical analysis was performed using unpaired *t*-test, *P < 0.05. Error bars indicate samples mean ± s.e.m. **c**, ELISA of TSLP protein from supernatant of proximal jejunal tissue explants after oral gavage at 2 h in *Pou2f3*^−*/*−^ or WT mice after overnight fasting. Biological replicates with total *N* = 28, pooled from 2 independent experiments. Statistical analysis was performed using ANOVA with correction for multiple comparisons, *P < 0.05. Error bars indicate samples mean ± s.e.m. NS, nonsignificant. **d**, Schematic of the protocol for measuring siLP ILC2 activation after feeding. Mice were fasted for 16 h overnight and given access to standard chow and water ad libitum (refed) or maintained on water control (fasted). Tissues were harvested 4 h later. **e**,**f**, Percentage of IL-13 (Smart13)^+^ ILC2 among ILC2s in proximal jejunal LP in *Tslpr*^*+*^^/+^Red5Smart13 or *Tslpr*^−*/*−^Red5Smart13 mice after 16-h fasting followed by 4-h refeeding ad libitum. **e**, Representative flowplots. **f**, Quantification. ILC2s were gated as Lin^−^CD45^+^IL-5 (Red5)^+^ cells. Biological replicates with total *N* = 31, pooled from at least 2 independent experiments. Statistical analysis was performed using ANOVA with correction for multiple comparisons, **P < 0.01, ***P < 0.001. Error bars indicate samples mean ± s.e.m. **g**, Percentage of IL-13 (Smart13)^+^ ILC2s among ILC2s in proximal jejunal LP in *Tslpr*^*fl/fl*^*Il5*^*Cre+*^(Red5)Smart13 mice after 16-h fasting followed by 4-h water or refeeding ad libitum. ILC2s were gated as Lin^−^CD45^+^IL-5 (Red5)^+^ cells. Biological replicates *N* = 8 each column group, pooled from 2 independent experiments. Statistical analysis was performed using unpaired *t*-test. Error bars indicate samples mean ± s.e.m. **h***,***i**, Percentage of IL-13 (Smart13)^+^ ILC2s among total ILC2s in proximal jejunal LP 4 h after refeeding ad libitum in overnight fasted *Tslp*^*fl/fl*^*Pdgfrα*^*CreERT2+*^Smart13 or littermate control *Pdgfrα*^*+/+*^Smart13 mice (ctrl) post-tamoxifen. **h**, Representative flowplots. **i**, Quantitation. ILC2s were gated on Lin^−^CD45^+^GATA3^+^ cells. Biological replicates *N* = 6 for control group and *N* = 13 for experiment group, pooled from at least 2 independent experiments. Statistical analysis was performed using unpaired *t*-test, *P < 0.05. Error bars indicate samples mean ± s.e.m. **j**, Percentage of IL-13 (Smart13)^+^ ILC2s among ILC2s in proximal jejunal LP 4 h after water or refeeding ad libitum in overnight fasted *Tslp*^*fl/fl*^*Lyve*^*Cre+*^Smart13 mice. ILC2s were gated on Lin^−^CD45^+^GATA3^+^ cells. Biological replicates *N* = 4 in each column group. Statistical analysis was performed using unpaired *t*-test, *P < 0.05. Error bars indicate samples mean ± s.e.m. Illustrations in **a** created with BioRender.com.

**Extended Data Fig. 3:**
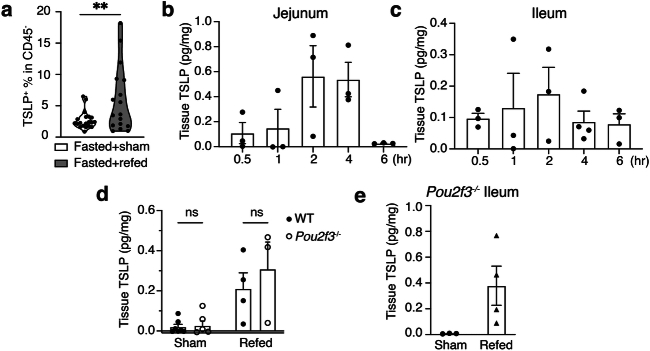
Feeding increases intestinal TSLP. **a-e**, Feeding increases intestinal TSLP. **a**, Percentage of TSLP-tdTomato^+^ cells among CD45^−^ cells in ileal lamina propria by flow cytometric analysis in fasted mice 2 hr after oral gavage of food (refed) or water as volumetric control (sham). Biological replicates N = 20 for control group and N = 17 for refeeding group. Statistic analysis was performed using unpaired t test, **P < 0.01. Error bars indicate samples mean ± SEM. **b**, ELISA of TSLP protein recovered from proximal jejumal tissue homogenates after oral gavage of food at designated times in fasted wild type (WT) mice. Biological replicates N = 3 per time point. Error bars indicate samples mean± SEM. **c**, ELISA of TSLP protein recovered from distal ileal tissue homogenates from fasted WT mice 2 hr after oral food gavage. Biological replicates N = 3 - 4 per time point. Statistical analysis was performed using ANOVA with correction for multiple comparisons. Error bars indicate samples mean ≥ SEM. **d**, ELISA of TSLP protein recovered from proximal jejunal tissue homogenates after oral gavage 2 hr in *Pou2f3*^−*/*−^ or WT mice after overnight fasting. Biological replicates N = 3 – 7 each column group. Error bars indicate samples mean ± SEM. **e**, ELISA of TSLP protein recovered from distal ileal tissue homogenates after oral gavage 2 hr in *Pou2f3*^−*/*−^ or WT mice after overnight fasting. Biological replicates N = 3 - 4 each column group. Error bars indicate samples mean ± SEM.

Feeding activates type 2 cytokine expression by small intestine ILC2s independently of the light–dark cycle^19^. After 16-h fasting, Flare-TSLP mice were orally gavaged with a 500-μl slurry of powdered diet containing ~2 kcal mixed nutrients (about 1% total daily caloric intake with components proportional to standard chow diet) or water control (Fig. 2a). After 2 h, the prevalence of TSLP-tdTomato^+^ CD45^−^ nonepithelial cells increased in proximal and distal siLP (Fig. 2b and Extended Data Fig. 3a). We confirmed TSLP protein upregulation by enzyme-linked immunosorbent assay (ELISA) in supernatants from small intestine tissue explants and homogenates, observing that TSLP amounts increased and peaked around 2 hr after gavage (Fig. 2c and Extended Data Fig. 3b,c). Notably, TSLP upregulation at this timepoint was unchanged after nutrient gavage of fasted *Pou2f3*^−/−^ mice, which lack tuft cells, indicating that tuft cells were not required for this increase (Fig. 2c and Extended Data Fig. 3d,e). To investigate the role for TSLP in ILC2 activation after feeding, we utilized cytokine reporter mice^20^ with marker alleles for IL-5 (Red5) and IL-13 (Smart13) crossed to TSLPR-deficient (*Tslpr*^−/−^) mice. We analyzed ILC2s from proximal siLP after 16-h fasting and 4-h refeeding with chow diet and water ad libitum or water alone (Fig. 2d). Consistent with previous findings^19^, ILC2s were activated 4 h after feeding, as evidenced by an increase in proportion of IL-13^+^ (Smart13)^+^ ILC2s that was attenuated in TSLPR-deficient mice (Fig. 2e,f). Furthermore, we generated ILC2 deletion of the TSLP receptor by crossing *Tslpr*^*fl/fl*^ mice with *Il5*^*Cre+*^ mice and confirmed that the loss of TSLPR expression on ILC2s impaired their activation after feeding (Fig. 2g).

We next sought to show the contributions of stromal-derived TSLP to activation of intestinal ILC2s by food intake. To achieve this, we generated *Tslp*^*fl/fl*^*Pdgfrα*^*CreERT2*^Smart13 and *Tslp*^*fl/fl*^*Lyve1*^*Cre*^Smart13 reporter mice, which delete TSLP from fibroblasts and LECs, respectively. Following administration of tamoxifen to *Tslp*^*fl/fl*^*Pdgfrα*^*CreERT2*^Smart13 mice, the loss of TSLP from fibroblasts resulted in attenuation of ILC2 activation after feeding, as indicated by diminished expression of the IL-13 reporter (Fig. 2h,i). In contrast, ILC2 activation in *Tslp*^*fl/fl*^*Lyve*^*Cre+*^*Smart13* mice remained intact (Fig. 2j). Thus, fibroblasts, rather than LECs, are the primary source of TSLP regulating ILC2 activation after feeding.

## GLP-2 mediates upregulation of TSLP in subepithelial fibroblasts

**Fig. 3:**
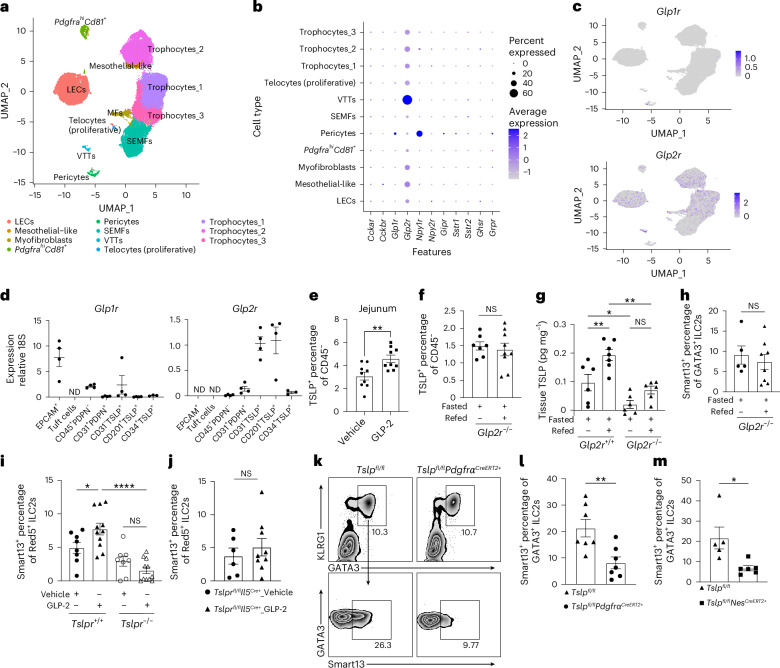
GLP-2 drives ILC2 activation through TSLP signaling. **a**–**c**, scRNA-seq analysis of sorted TSLP-tdTomato^+^ cells from the siLP. **a**, UMAP representing cell clustering analysis. **b**, Expression of gut hormorne receptors by TSLP-tdTomato ^+^ stromal cells. **c**, Feature plots representing *Glp1r* (top) and *Glp2r* (bottom) expression. **d**, RT–qPCR analysis of *Glp1r* (left) and *Glp2r* (right) expression on sorted gut. CD45^−^EPCAM^+^ (epithelial cells), tuft cells, CD45^+^PDPN^−^ (hematopoietic cells), CD45^−^CD31^+^PDPN^+^ (LECs), CD45^−^CD31^−^TSLP-tdTomato^+^ total stromal cells, CD45^−^CD31^−^TSLP-tdTomato^+^ CD201^+^ (telocytes) and CD45^−^CD31^−^TSLP-tdTomato^+^ CD34^+^ (trophocytes). Biological replicates *N* = 3 or 4, pooled from 2 independent experiments. Error bars indicate samples mean ± s.e.m. **e**, Percentage of TSLP-tdTomato^+^cells among CD45^−^ cells in proximal jejunal LP after three consecutive GLP-2[Gly2] s.c. injections as assessed by flow cytometric analysis. Biological replicates *N* = 9 for each column group, pooled from at least 2 independent experiments. Statistical analysis was performed using unpaired *t*-test, **P < 0.01. Error bars indicate samples mean ± s.e.m. **f**, Flow cytometric analysis showing percentage of total TSLP-tdTomato^+^ cells among CD45^−^ cells in proximal jejunal LP from fasted *Glp2r*^−*/*−^Flare-TSLP mice after oral food gavage at 2 h. Biological replicates *N* = 7 for control group and *N* = 9 for refeeding group, pooled from at least 2 independent experiments. Statistical analysis was performed using unpaired *t*-test. Error bars indicate samples mean ± s.e.m. **g**, ELISA of TSLP protein from supernatant of proximal jejunal tissue explants after oral gavage at 2 h in *Glp2r*^−*/*−^ or WT mice after overnight fasting. Biological replicates with total *N* = 25, pooled from at least 2 independent experiments. Statistical analysis was performed using ANOVA with correction for multiple comparisons, *P < 0.05, **P < 0.01. Error bars indicate samples mean ± s.e.m. **h**, Quantification of percentage of IL-13 (Smart13)^+^ ILC2s among proximal jejunal LP ILC2s from fasted *Glp2r*^−*/*−^Smart13 mice after 16-h fasting followed by 4-h water or refeeding ad libitum. ILC2s were gated on Lin^−^CD45^+^GATA3^+^ cells. Biological replicates *N* = 5 for control group and *N* = 8 for refeeding group, pooled from at least 2 independent experiments. Statistical analysis was performed using unpaired *t*-test. Error bars indicate samples mean ± s.e.m. **i**, Quantification of percentage of IL-13 (Smart13)^+^ ILC2s among proximal jejunal LP ILC2s in *Tslpr*^*+/+*^Red5Smart13 or *Tslpr*^−*/*−^Red5Smart13 mice after three daily injections of GLP-2[Gly2]. ILC2s were gated on Lin^−^CD45^+^IL-5(Red5)^+^ cells. Biological replicates with total *N* = 38, pooled from at least 2 independent experiments. Statistical analysis was performed using ANOVA with correction for multiple comparisons, *P < 0.05, ****P < 0.0001. Error bars indicate samples mean ± s.e.m. **j**, Quantification of percentage of IL-13 (Smart13)^+^ ILC2s among proximal jejunal LP ILC2s in *Tslpr*^*fl/fl*^*Il5*^*Cre+*^ Smart13 after three daily injections of GLP-2[Gly2] or vehicle control. ILC2s were gated on Lin^−^CD45^+^IL-5(Red5)^+^ cells. Biological replicates *N* = 6 for vehicle control group and *N* = 9 for GLP-2[Gly2] injection group, pooled of 2 independent experiments. Statistical analysis was performed using unpaired *t*-test. Error bars indicate samples mean ± s.e.m. **k**,**l**, Flow cytometric analysis of percentage of IL-13 (Smart13)^+^ ILC2s among proximal jejunal LP ILC2s in *Tslp*^*fl/fl*^*Pdgfrα*^*CreERT2+*^Smart13 or littermate *Tslp*^*fl/*^^fl^ Smart13 controls post-tamoxifen followed by three daily injections of GLP-2[Gly2]. **k**, Representative flowplots. **l**, Quantitation. ILC2s were gated as Lin^−^CD45^+^GATA3^+^ cells. Biological replicates *N* = 7 for each column group, pooled from at least 2 independent experiments. Statistical analysis was performed using unpaired *t*-test, **P < 0.01. Error bars indicate samples mean ± s.e.m. **m**, Flow cytometric analysis of percentage of IL-13 (Smart13)^+^ ILC2s among proximal jejunal LP ILC2s in *Tslp*^*fl/fl*^*Nes*^*CreERT2+*^Smart13 or littermate *Tslp*^*fl/*^^fl^ Smart13 controls post-tamoxifen followed by three daily injections of GLP-2[Gly2]. Biological replicates *N* = 6 for control group and *N* = 5 for experiment group, pooled from 2 independent experiments. Statistical analysis was performed using unpaired *t*-test, *P < 0.05. Error bars indicate samples mean ± s.e.m.

**Extended Data Fig. 4:**
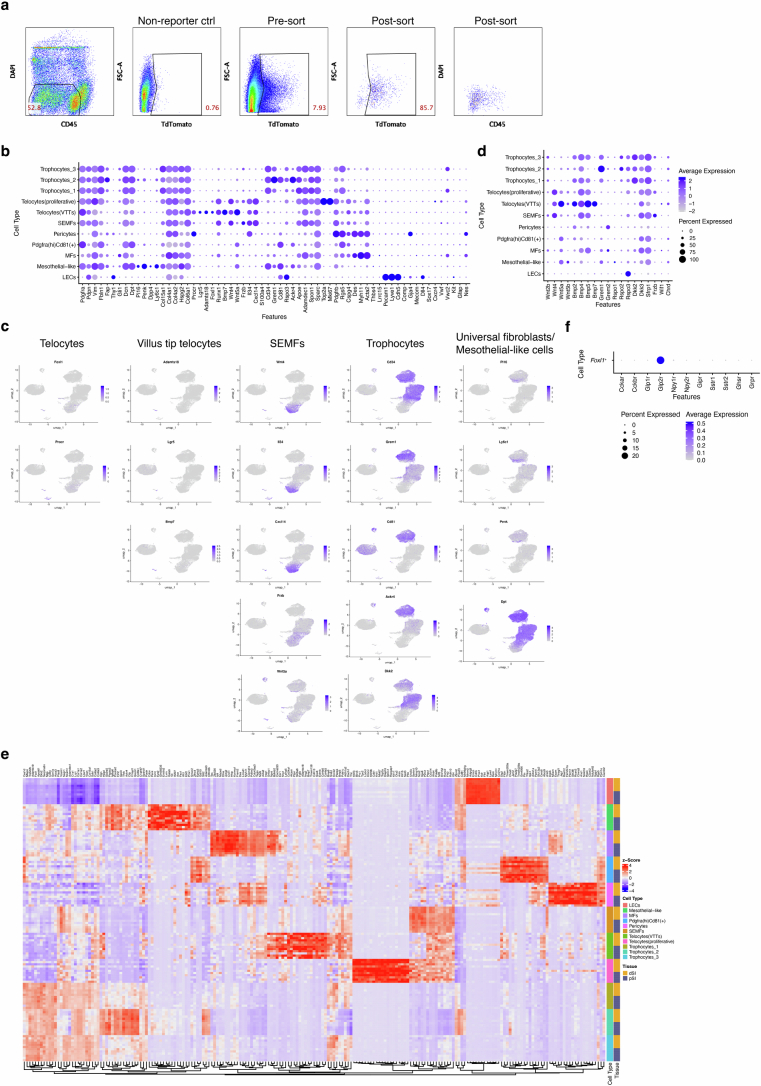
Single cell RNA sequencing (scRNA-seq) analyses of TSLP-Tdtomato^+^ cells from mouse small intestinal lamina propria (siLP). **a**, Gating strategy for FACS sorting of TSLP- tdTomato^+^ cells from intestinal LP of Flare-TSLP mice. **b-f**, scRNA-seq analysis of pooled proximal and distal siLP TSLP- tdTomato^+^ cells. **b**, Expression levels of marker genes for annotated cell types. **c**, Feature plots represent marker genes for telocytes, VTTs, SEMFs, trophocytes and universal fibroblasts/mesothelial-like cells. **d**, Expression levels of WNT and BMP signaling molecules for annotated cell types. **e**, Heatmap representation of top 20 most significantly changed genes including both directions from differential gene expression (DE) analysis between cell types in the proximal and distal small instestine. Columns were manually ordered by cell type. Plot shows z-scores. **f**, Expression levels of gut hormone receptors in *Foxl1+* telocytes.

**Extended Data Fig. 5:**
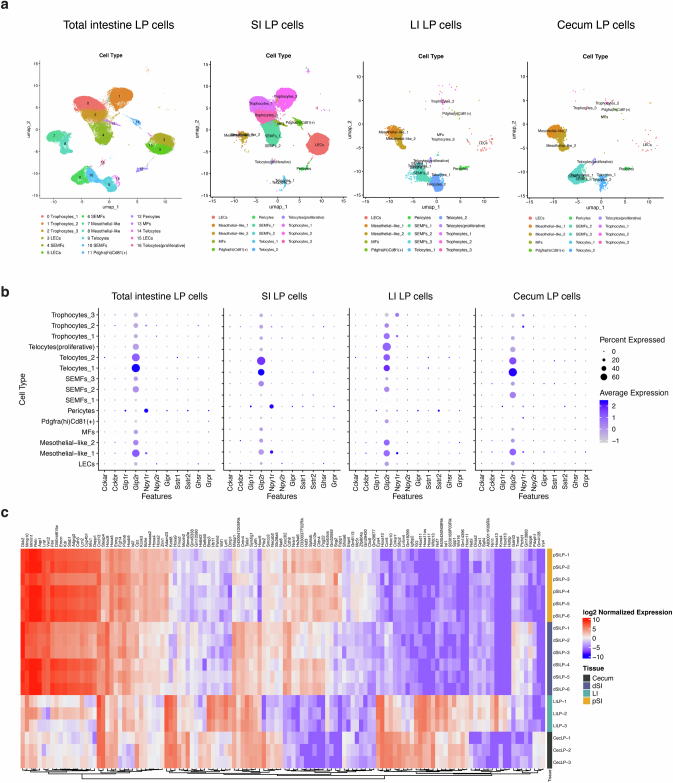
scRNA-seq analyses of TSLP-Tdtomato^+^ cells from mouse intestinal lamina propria (continued). **a**, UMAPs representing cell clustering in pooled cells (total intestine LP cells), siLP, LILP and cecum LP samples. Unsupervised cell clustering analysis revealed 17 clusters based on differential gene expression. Annotation was conducted according to dominant cluster-specific genes, while also considering existing stromal subsets. Subsets of fibroblasts were categorized as SEMFs (telocytes_1, telocytes_2, SEMFs_1, SEMFs_2), trophocytes (trophocytes_1, trophocytes_2, trophocytes_3), and recently identified *Pi16*^*+*^ universal fibroblasts or mesothelial-like cells (mesothelial-like_1, mesothelial-like_2). We also identified myofibroblasts (MFs), and an undefined cluster characterized by *Pdgfra*^*hi*^ *CD81*^*+*^*Ptn*^*+*^ fibroblasts. Additional cells included LECs and pericytes. Some cell types were enriched in specific tissues; for instance, trophocytes, *Pdgfra*^*hi*^ *CD81*^*+*^cells, and LECs were primarily found in small intestine samples, while SEMFs_2 were more abundant in the large intestine and cecum but not in small intestine. Telocytes_2 were mostly enriched in the large intestine with few minimal in cecum and none in small intestine. **b**, Gut hormone receptor expression in total pooled intestine LP cells, LILP and cecum LP samples. **c**, Heatmap representing top 20 fold-change genes including both directions between tissue comparisons in DE analysis. Plot shows log2 normalized expression. The transcriptomic signatures of TSLP-tdTomato^+^ cells were similar betwen proximal and distal small intestine, as well as between large intestine and cecum.

To better understand the cells underlying feeding-induced TSLP-ILC2 signaling, we performed single-cell RNA sequencing (scRNA-seq) of TSLP-Tdtomato^+^ cells isolated from the proximal and distal siLP of Flare-TSLP mice using at least three biological samples from each tissue (Extended Data Fig. 4a). Unsupervised cell clustering analysis revealed the main stromal cell types were fibroblasts and LECs, and consistent with the flow cytometric analyses (Fig. 3a and Extended Data Figs. 4b and 5a). Principal subsets of fibroblasts included subepithelial myofibroblasts (SEMFs) (which include *Foxl1*^*+*^ telocytes), *Lgr5*^*+*^ VTTs, *Mik67*^+^ proliferative telocytes, *Grem1*^*+*^ trophocytes and recently described *Pi16*^*+*^ ‘universal’ fibroblasts with transcriptomic signatures of mesothelial-like cells^21^ (Fig. 3a and Extended Data Fig. 4b,c). In accordance with their anatomic locations, SEMFs and trophocytes were highlighted by expression of distinct WNTs and BMPs (Extended Data Fig. 4d). SEMFs were enriched for noncanonical WNTs (such as *Wnt4* and *Wnt5a*) and *Bmps*, and typically expressed by telocytes. Notably, *Bmp7* is specifically enriched in VTTs. In contrast, trophocytes were enriched for canonical WNTs, such as *Wnt2b*, and BMP inhibitors, including *Grem1*, *Rspo3* and *Dkk2*. The three trophocyte subsets exhibited similar transcriptomic signatures, as did SEMFs, VTTs and proliferative telocytes (Extended Data Fig. 4e). These cell types expressed comparable gene signatures across the proximal and distal regions of small bowel (Extended Data Figs. 4e and 5c).

**Extended Data Fig. 6:**
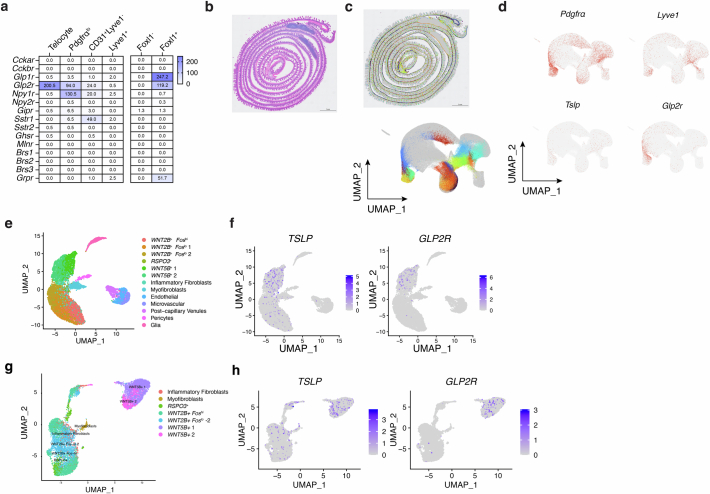
scRNA-seq analyses of publicly available mouse and human intestinal stromal cell datasets. **a**, Heatmaps representing expression levels of gut hormone receptors in PDGFRa^hi^ telocytes^15^, PDGFRa^lo^ trophocytes^15^, CD31^+^LYVE1^−^ endothelial cells^15^, LYVE1^+^ LECs^15^, *Foxl1*^+^ telocytes^16^ and *Foxl1*^−^ stromal cells^16^ from mouse datasets. **b-d**, 10x Genomics published Visium mouse small intestine dataset^59^. **b**, Hematoxylin and eosin staining of the intestinal tissue specimen. **c**, Color-coded cell clusters in the lamina propria (LP) shown *in situ* (**top**) and on a UMAP (**bottom**). **d**, UMAP feature plots representing gene expression level and distribution of *Pdgfra*, *Lyve1*, *Tslp* and *Glp2r*. *Tslp* and *Glp2r* expression enriched in the stromal populations of intestinal LP. **e-h**, Analyses of human intestinal datasets^24,25^. **e**, UMAP representing cell clustering of human dataset^24^. **f**, Feature plots of *TSLP* (**left**) and *GLP2R* (**right**) expression levels. **g**, UMAP representing cell clustering of human dataset^25^. **h**, Feature plots of *TSLP* (**left**) and *GLP2R* (**right**) expression levels.

Nutrients in food are absorbed primarily by the prevalent enterocytes and sensed by less frequent EECs, which respond to luminal content by releasing peptide hormones that regulate nutrient uptake, metabolism, intestinal motility and satiety. EECs, named by dominant hormones they release, develop along vertically zonated villus trajectories through nodes of differentiation accompanied by activation of overlapping components of their peptide hormone repertoires^22,23^. We next investigated the expression of gut peptide hormone receptors expressed by subepithelial fibroblasts that would enable crosstalk between nutrient-sensing EECs and these structural cells. Our data, consistent with that of others, revealed that intestinal stromal cells express high amounts of mRNA for the receptor for glucagon-like peptide-2 (GLP-2) with less consistent evidence for other gut peptide hormone receptors (Fig. 3b,c), in both mouse and human^15,16,24,25^ (Extended Data Fig. 6a,e–h). Spatial transcriptional analysis from a public dataset of mouse small intestine revealed that *Glp2r* expression associated predominantly with *Pdgfrα*^*hi*^ populations that also co-expressed *Tslp* (Extended Data Fig. 6b–d). Of note, *Foxl1*^*+*^ telocytes, including VTTs, were enriched strongly for *Glp2r* expression (Fig. 3b and Extended Data Fig. 4f).

**Extended Data Fig. 7:**
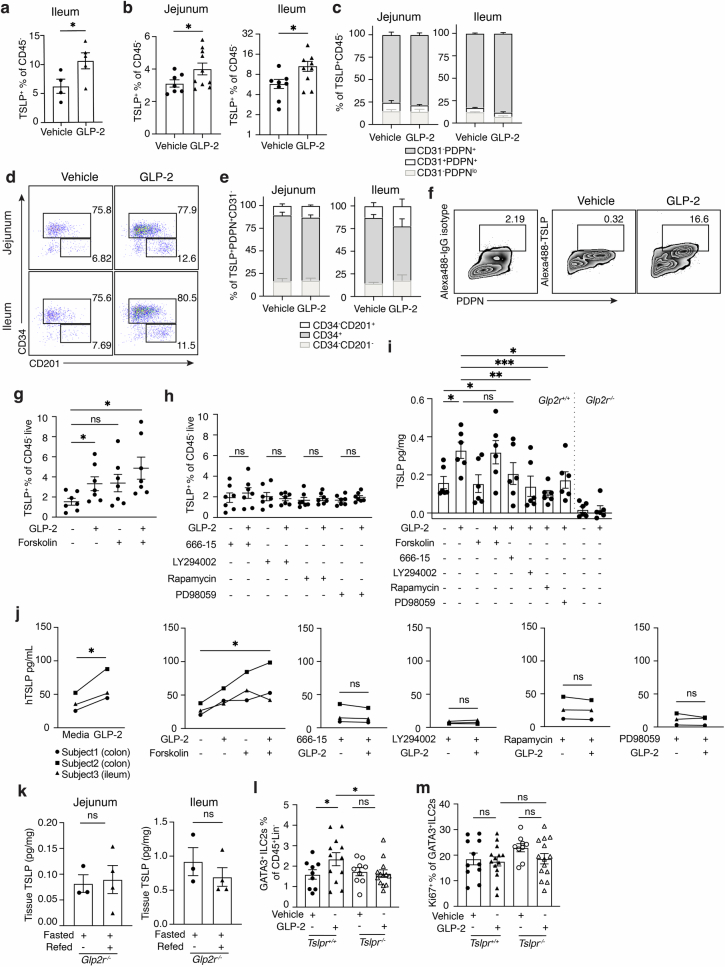
L cell GLP-2 drives GLP-2R- and TSLP-dependent ILC2 activation. **a**, Flow cytometric analysis of TSLP-tdTomato^+^ cells in ileal lamina propria (LP) after 3 daily GLP-2[Gly2] injections. Biological replicates N = 4-5 for each group. **b-e** Flow cytometric analysis of TSLP-tdTomato^+^ cells in jejunal and ileal lamina propria (LP) at 2 hr after GLP-2[Gly2] administration. **b**, Percentage of total TSLP-tdTomato^+^ cells among CD45^−^ cells in jejunal (**left**) and ileal LP (**right**). Biological replicates N = 7-10 (left) and N = 8-9 (right) for each group. **c**, Proportion of CD31^+^PDPN^+^ LECs, CD31^−^PDPN^+^ and CD31^−^PDPN^lo^ stromal populations within TSLP-tdTomato^+^ cells in jejunum (**left**) and in ileum (**right**). Biological replicates N = 3-4 each group. **d-e**, Characterization of CD201^+^ and CD34^+^ cells within CD45^−^ PDPN^+^CD31^−^TSLP-tdTomato^+^ stromal cells. **d**, Representative flow cytometric analysis. **e**, Quantification. Biological replicates N = 3-4 each column group. **f**, Intracellular antibody staining for TSLP protein in sorted CD45^−^CD31^−^TSLP-tdTomato^+^ stromal cells from small intestinal LP 4 hr after incubation with 10 µg/ml GLP-2[Gly2] with Brefeldin A *in vitro*. **g-h**, Mouse fibroblasts treated with agonists (**g**) and/or inhibitors (**h**). TSLP levels were assessed by flow cytometric analysis using frequency of TSLP^+^ cells among total cultured fibroblasts characterized by CD45^−^PDPN^+^. Biological replicates N = 7 each group. **i**, Mouse intestinal explants treated with agonists and/or inhibitors. TSLP protein levels in the supernatants were measured by ELISA. Biological replicates total N = 77. **j**, TSLP protein levels in supernatants of human fibroblast cultures treated with agonists and/or inhibitors. Paired t-tests were performed for statistical analysis. Biological replicates N = 3. **k**, TSLP protein recovered from jejunal and ileal tissue homogenates from fasted *Glp2r*^−*/*−^ mice 2 hr after food gavage. Biological replicates N = 3-4 each group. **l-m**, Flow cytometric analysis of ILC2s in jejunal LP in *Tslpr*±^*+*^ and *Tslpr*^−*/*−^ mice after 3 daily injections of GLP-2[Gly2]. **l**, Percentage of ILC2s among Lin^−^CD45^+^ cells. **m**, Quantification of percentage of Ki67^+^ cells among ILC2s. ILC2s were gated on CD45^+^Lin^−^GATA3^+^ cells. Biological replicates N = 9-14 for each group. Graphs represent data pooled from at least two experiments. Error bars indicate samples mean± SEM. Unless specified, upaired t-test was used for analyses of difference between 2 groups. ANOVA test was used for analyzing experiments with >2 groups adjusted for multiple comparisons. *P < 0.05, **P < 0.01, ***P < 0.001.

Although prominent in pancreatic glucagon-producing alpha cells, proglucagon is also expressed highly in subsets of small intestinal EECs, particularly L cells, which express tissue-specific convertases that post-translationally process proglucagon to the peptides glucagon-like peptide-1 (GLP-1), GLP-2 and oxyntomodulin rather than glucagon^26–28^. In response to food constituents, proglucagon-derived peptides are secreted into the basolateral space, where the activities of GLP-1 and GLP-2 are kept localized by high concentrations of the inactivating dipeptidyl peptidase-4 enzyme. GLP-2 is intestinotrophic, promoting microvillus lengthening, increasing vascular flow and enhancing the epithelial barrier, in part by stimulating release of insulin-like growth factor-1 and epidermal growth factor from subepithelial fibroblasts^26^. Using qPCR analysis, we detected *Glp2r* expression by stromal cells but not epithelial cells (Fig. 3d). Further, administration of stabilized GLP-2R agonist, GLP-2[Gly2], significantly increased the prevalence of TSLP-tdTomato^+^ cells in siLP compared to mice treated with vehicle control (Fig. 3e and Extended Data Fig. 7a,b). The proportions of fibroblasts or LECs were not altered by GLP-2[Gly2] administration (Extended Data Fig. 7c), but there appeared to be more CD201^+^ telocytes within the CD45^−^TSLP-tdTomato^+^CD31^−^ stromal populations in the siLP (Extended Data Fig. 7d,e). In vitro incubation of sorted CD45^−^TSLP-tdTomato^+^CD31^−^ cells with GLP-2[Gly2] resulted in increased antibody labeling of TSLP protein compared to vehicle control-treated cells (Extended Data Fig. 7f).

In considering how GLP-2 might induce TSLP expression in the gut subepithelial fibroblasts, we note previous studies implicating GLP-2 stimulation of enteric neurons by signals through PI3K with mTOR or ERK to induce VIP expression^29^. GLP-2 can also increase intracellular cyclic AMP concentrations, including in fibroblasts^30,31^, and cAMP has been implicated in TSLP production and release^32,33^. To investigate the potential mechanisms by which GLP-2 regulates TSLP upregulation, we measured TSLP protein concentrations in three experimental settings, including primary intestinal fibroblasts derived from mouse and human tissues as well as ex vivo mouse intestinal explants. Fibroblast monolayers or tissue explants were treated with GLP-2 alone or in combination with a cAMP agonist forskolin or with inhibitors targeting CREB (666-15), PI3K (LY294002), mTOR (rapamycin), or MEK/ERK (PD98059) (Extended Data Fig. 7g–j). Treatment with GLP-2 agonist alone significantly increased TSLP amounts, whereas cotreatment with PI3K, mTOR and MEK/ERK inhibitors blocked the increased TSLP in all three models, suggesting the engagement of PI3K-mTOR and PI3K-ERK signaling pathways, and consistent with previous findings. In mouse and human fibroblasts, forskolin increased TSLP and antagonism of CREB using 666-15 abrogated GLP-2-induced TSLP upregulation (Extended Data Fig. 7g,h,j); mouse explants were less affected by interruption of these pathways (Extended Data Fig. 7i). Together, these data suggest that GLP-2 regulates TSLP upregulation through PI3K-mTOR and PI3K-ERK signaling with the possible involvement of the cAMP-CREB axis in TSLP induction.

## GLP-2 mediates TSLP-dependent activation of intestinal ILC2s in vivo

In support of the role for GLP-2 in the induction of TSLP by feeding, the increased prevalence of TSLP-tdTomato^+^ cells in proximal siLP and the amounts of TSLP protein recovered from intestinal tissue explants and homogenates were abrogated in fasted and refed mice lacking GLP-2R (Fig. 3f,g and Extended Data Fig. 7k). Activation of ILC2s after feeding was also attenuated in *Glp2r*^−/−^ mice as assessed by IL-13 reporter (Smart13) expression (Fig. 3h). These results suggest that both TSLP production and ILC2 activation are downstream effects of GLP-2 induced by feeding. Administration of GLP-2[Gly2] led to an increase in activated IL-13-reporter^+^ ILC2s that was not associated with proliferation and that was attenuated in TSLPR-deficient mice (Fig. 3i and Extended Data Fig. 7l,m), as well as in *Tslpr*^*fl/fl*^*Il5*^*Cre+*^Smart13 mice (Fig. 3j), which lack TSLPR on ILC2s, and tamoxifen-treated *Tslp*^*fl/fl*^*Pdgfrα*^*CreERT2+*^Smart13 mice (Fig. 3k,l), which lack TSLP in fibroblasts.

**Extended Data Fig. 8:**
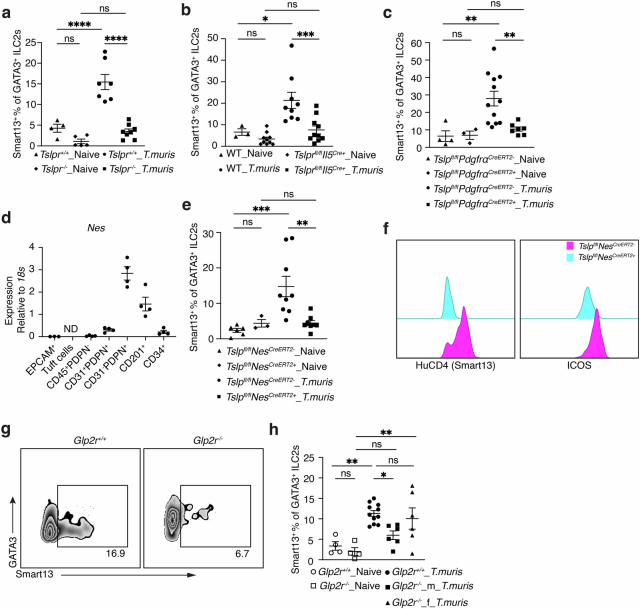
*T. muris* infection drives stromal TSLP-dependent ILC2 activation. **a-c**, Flow cytometric analysis of ILC2s in large intestinal lamina propria (LILP) on day 21 post *T. muris* infection. Percentage of IL-13 (Smart13)^+^ ILC2 among ILC2s in LI LP in *Tslpr*^−*/*−^ or *Tslpr*±^*+*^ mice, biological replicates N = 4-8 each column group, ****P < 0.0001 (**a**), *Tslpr*^*fl/fl*^*Il5*^*Cre+*^ or WT controls, biological replicates N = 3-10 each column group, *P < 0.05, ***P < 0.001 (**b**), and *Tslp*^*fl/fl*^*Pdgfra*^*CreERT2+*^ or *Tslp*^*fl/fl*^ controls, biological replicates N = 3-12 each column group, **P < 0.01 (**c**). ILC2s were gated on Lin^−^CD45^+^GATA3^+^ cells. **d**, RT-qPCR analysis for *Nes* expression in sorted cells from the small intestine, including CD45^−^EPCAM^+^epithelial cells, tuft cells, CD45^+^PDPN^−^ hematopoietic cells, CD45^−^CD31^+^PDPN^+^ LECs, CD45^−^CD31^−^PDPN^+^, CD45^−^CD31^−^CD201^+^, and CD45^−^CD31^−^CD34^+^ stromal cells. Biological replicates N = 3-4 each column group. **e-f**, Flow cytometric analyses of LILP ILC2s in *Tslp*^*fl/fl*^*Nes*^*CreERT2+*^ or control mice on 21 d.p.i. Percentage of IL-13 (Smart13)^+^ ILC2 among ILC2s in LI LP in *Tslp*^*fl/fl*^*Nes*^*CreERT2+*^ or control (**e**) and the expression levels of ILC2s activation markers including HuCD4 (Smart13) and ICOS were lower in *Tslp*^*fl/fl*^*Nes*^*CreERT2+*^ mice than controls (**f**). ILC2s were gated on Lin^−^CD45^+^GATA3^+^ cells. Biological replicates N = 3-9 each column group, **P < 0.01, ***P < 0.001. **g-h**, Flow cytometric analysis of LILP ILC2s in *Glp2r*^−*/*−^ and *Glp2r*±^*+*^ mice 21 d.p.i. Flow charts represent IL-13 (Smart13) levels (**g**) and percentage of IL-13 (Smart13)^+^ ILC2s among total ILC2s in LI LP (**h**). ILC2s were gated on Lin^−^CD45^+^GATA3^+^ cells. Biological replicates N = 4-11 each column group, *P < 0.05, **P < 0.01. Data are representative of at least two independent experiments. Statistical analyses were performed using ANOVA with correction for multiple comparisons. Error bars indicate samples mean ± SEM.

TSLP is also important for type 2 immune responses after *T.* *muris* infection^12^. In line with this, the activation of *Tslpr*^−*/*−^ ILC2s was diminished significantly following *T.* *muris* infection (Extended Data Fig. 8a). A similar reduction in ILC2 activation was observed in *Tslpr*^*fl/fl*^*Il5*^*Cre+*^Smart13 mice (Extended Data Fig. 8b), and *Tslp*^*fl/fl*^*Pdgfrα*^*CreERT2+*^Smart13 after tamoxifen treatment (Extended Data Fig. 8c). Given that *Glp2r* expression was highly expressed in telocytes among stromal cell populations in the small intestine (Fig. 3b and Extended Data Fig. 5b), we hypothesize that these cells may represent the primary GLP-2-responsive cells mediating the downstream TSLP-ILC2 signaling axis. qPCR analysis revealed that *Nes*, the gene encoding for the intermediate filament protein Nestin, was expressed by total stromal cells, with CD201^+^ subepithelial villus fibroblasts including telocytes showing the highest amounts of *Nes* expression as compared to other stromal subsets, including CD34^+^ trophocytes (Extended Data Fig. 8d). We crossed *Tslp*^*fl/fl*^ Smart13 reporter mice with *Nes*^*CreERT2*^ mice to achieve deletion of *Tslp* in subepithelial villus fibroblasts. After tamoxifen treatment, *Tslp*^*fl/fl*^*Nes*^*CreERT2+*^Smart13 mice exhibited diminished ILC2 activation following GLP-2[Gly2] administration and this was phenocopied using *Tslp*^*fl/fl*^*Pdgfrα*^*CreERT2+*^Smart13 mice (Fig. 3m), indicating that subepithelial villus fibroblasts represent the main source of TSLP in the small intestine that mediates ILC2 activation downstream of GLP-2. Further, after *T.* *muris* infection, *Tslp*^*fl/fl*^*Nes*^*CreERT2+*^Smart13 mice also exhibited impaired ILC2 activation as indicated by the reduced percentage of Smart13^+^ ILC2s and lower expression of Smart13 and ICOS (Extended Data Fig. 8e,f), and consistent with enriched *Glp2r* expression by TSLP*-*tdTomato^+^ subepithelial fibroblasts, including telocytes, in the mouse cecum and proximal colon (Extended Data Fig. 5b) where the parasites reside. These data indicate that fibroblast-derived TSLP, particularly from the telocytes, is required for optimal activation of TSLPR^+^ ILC2s after food intake and *T.* *muris* infection. Of note, male *Glp2r*^−/−^ mice had diminished ILC2 activation compared to wild-type (WT) mice following *T.* *muris* infection, whereas female *Glp2r*^−/−^ mice were more variable (Extended Data Fig. 8g,h), and suggesting that GLP-2 may play a role in TSLP-dependent ILC2 activation during *T.* *muris* infection.

Taken together, these data support a biological pathway linking the activation of preproglucagon^+^ EECs with GLP-2-mediated stimulation of subepithelial fibroblasts leading to increased production of TSLP, which activates resident ILC2s to produce IL-13 in the intestine.

## GLP-2 mediates ILC2-dependent amplification of the small intestinal tuft cell circuit

**Fig. 4:**
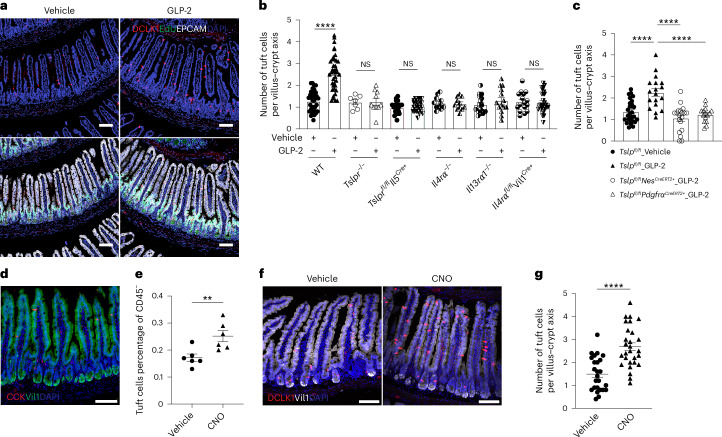
GLP-2 drives ILC2-dependent tuft cell expansion. **a**, Representative imaging of tuft cells in jejunum after three daily injections of GLP-2[Gly2]. EdU was injected 24 h before tissue harvest. Green, EdU; red, DCLK1; white, EPCAM; blue, DAPI; ×20 objective. Scale bars, 100 µm. **b**, Quantification of jejunal tuft cells after three daily GLP-2[Gly2] injections or vehicle control in WT, *Tslpr*^−/−^, *Tslpr*^*fl/fl*^*Il5*^*Cre+*^, *Il4rα*^−/−^, *Il13rα*^−/−^ and *Il4rα*^*fl/fl*^Vil1^Cre+^ mice. For tuft cell quantification, images were acquired using ×20 objective, and total DCLK1^+^EPCAM^+^ cells in each field were counted and normalized by total number of villus–crypt axes in the field. Each dot represents an imaging field, total *N* = 232; data pooled from 3 to 10 mice. A plot with averages of each mouse is included in Extended Data Fig. 9b. Statistical analysis was performed using ANOVA with correction for multiple comparisons, ****P < 0.0001. Error bars indicate samples mean ± s.e.m. **c**, Quantification of jejunal tuft cells after three daily GLP-2[Gly2] or vehicle control injections in *Tslp*^*fl/fl*^, *Tslp*^*fl/fl*^*Nes*^*CreERT2+*^ and *Tslp*^*fl/fl*^*Pdgfrα*^*CreERT2+*^ mice. Each dot represents an imaging field, total *N* = 80; data pooled from 3 to 6 mice. A plot with averages of each mouse is included in Extended Data Fig. 9d. Statistical analysis was performed using ANOVA with correction for multiple comparisons, ****P < 0.0001. Error bars indicate samples mean ± s.e.m. **d**, Representative imaging showing CCK^+^ EECs in *Vil1*^*Flp*^*Cck*^*Cre*^*R26*^*Dual*−*hM3Dq*^ mice. Red, *Cck*-mCherry; green, Vil1-GFP; blue, DAPI; ×20 objective. Scale bar, 100 µm. **e**–**g**, Quantification of jejunal tuft cells after administration of clozapine N-oxide CNO or vehicle control in *Vil1*^*Flp*^*Cck*^*Cre*^*R26*^*Dual*−*hM3Dq*^ mice. **e**, Percentage of tuft cells among the CD45^−^ epithelial fraction by flow cytometric analysis. Two independent experiment repeats were combined to generate the plot. Biological replicates *N* = 6 for each column group were compared using two-tailed unpaired *t*-test, **P < 0.005. Error bars indicate samples mean ± s.e.m. **f**, Representative imaging of tuft cells. Red, DCLK1; white, Vil1-GFP; blue, DAPI; ×20 objective. Scale bars, 100 µm. **g**, Quantification of tuft cells. Each dot represents an imaging field, *N* = 25 for control group and *N* = 28 for CNO treatment group; data pooled from 6 mice each group from 2 independent experiments. A plot with averages of each mouse is included in Extended Data Fig. 9g. Statistical analysis was performed using two-tailed unpaired *t*-test, ****P < 0.0001. Error bars indicate samples mean ± s.e.m.

**Extended Data Fig. 9:**
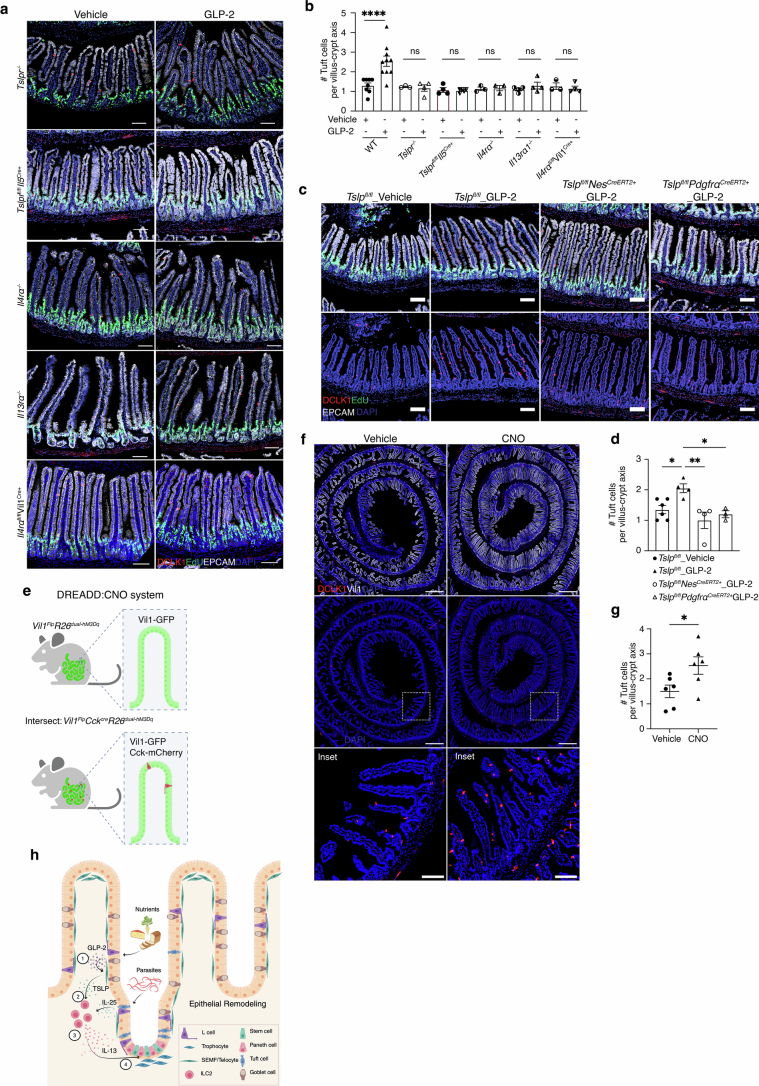
GLP-2 drives ILC2-dependent amplification of the tuft cell circuit. **a**, Representative imaging of jejunal tuft cells in *Tslpr*^−/−^, *Tslpr*^*fl/fl*^*Il5*^*Cre+*^*, Il4ra*^−/−^, *Il13ra1*^−/−^ and *Il4ra*^*fl/fl*^Vil1^Cre+^ mice. Green, EdU; Red, DCLK1; White, EPCAM; Blue, DAPI. 20x objective. Scale bar, 100 µm. **b**, Quantification of jejunal tuft cells after 3 daily GLP-2[Gly2] injections in WT, *Tslpr*^−/−^, *Tslpr*^*fl/fl*^*Il5*^*Cre+*^*, Il4ra*^−/−^, *Il13ra*^−/−^ and *Il4ra*^*fl/fl*^Vil1^Cre+^ mice. For tuft cell quantification, images were acquired using 20x objective and total DCLK1^+^EPCAM^+^ cells in each field were counted and normalized by total number of villus-crypt axes in the field. Each dot represents a mouse, N = 3-10 each column. Statistical analysis was performed using ANOVA with correction for multiple comparisons, ****P < 0.0001. Error bars indicate samples mean± SEM. **c**, Representative imaging of jejunal tuft cells in *Tslp*^*fl/fl*^, *Tslp*^*fl/fl*^*Nes*^*CreERT2+*^, and *Tslp*^*fl/fl*^*Pdgfra*^*CreERT2+*^mice. Green, EdU; Red, DCLK1; White, EPCAM; Blue, DAPI. 20x objective. Scale bar, 100 µm. **d**, Quantification of jejunal tuft cells after 3 daily GLP-2[Gly2] injections in *Tslp*^*fl/fl*^*, Tslp*^*fl/fl*^*Nes*^*CreERT2+*^, and *Tslp*^*fl/fl*^*Pdgfra*^*CreERT2+*^ mice. Each dot represents a mouse, N = 3-6. Statistical analysis was performed using ANOVA with correction for multiple comparisons, *P < 0.05, **P < 0.01. Error bars indicate samples mean± SEM. **e**, Schematic illustrating the *Vil1*^*Flp*^*Cck*^*Cre*^*R26*^*Dual*−*hM3Dq*^ DREADD and CNO system. **f-g**, Quantification of jejunal tuft cells in *Vil1*^*Flp*^*Cck*^*Cre*^*R26*^*Dual*−*hM3Dq*^ mice after administration of clozapine N-oxide (CNO) or vehicle control. Each dot represents a mouse. **f**, Representative imaging of tuft cells. Red, DCLK1; White, *Vil1*-GFP; Blue, DAPI. 20x objective. Scale bar, 100 µm. **g**, Quantification of tuft cells. Two experiment repeats, each dot represents a mouse, N = 6 for each column group. Statistical analysis was performed using two-tailed unpaired t-test,*P < 0.05. Error bars indicate samples mean± SEM. **h**, Proposed model for the mechanism of GLP-2 – TSLP – ILC2 signaling pathway in promoting intestine tissue remodeling. GLP-2 from L cells (**1**) stimulates telocytes to produce TSLP (**2**) that activates ILC2s (**3**), thus linking nutrient ingestion and IL-13-mediated amplification of epithelial tuft cells. (**4**) *N. brasiliensis* short circuits the physiologic pathway by directly stimulating tuft cells to release IL-25 that activates ILC2s and drives the ILC2–tuft cell circuit amplification. Panels **e** and **h** created using BioRender.com.

Activation of intestinal ILC2s to release IL-13 leads to biased production of tuft cells from the transit-amplifying zone, which depends on IL-4Rα expression on intestinal epithelial cells^6,7^. Consistent with these findings, administration of GLP-2[Gly2] resulted in increased numbers of small intestinal tuft cells independent of proliferation as indicated by 5-ethynyl-2′-deoxyuridine (EdU) negative staining, which was significantly attenuated in *Tslpr*^−/−^, *Tslpr*^*fl/fl*^*Il5*^*Cre+*^, *Il13rα1*^−/−^ and *Il4rα*^−/−^ mice (Fig. 4a,b and Extended Data Fig. 9a,b). Further, the increase in tuft cells was diminished in *Il4ra*^*fl/fl*^Vil1^Cre+^ mice, supporting a role for epithelial IL-4Rα in activating the circuit (Fig. 4b and Extended Data Fig. 9a,b). Furthermore, GLP-2-induced tuft cell hyperplasia was attenuated in tamoxifen-treated *Tslp*^*fl/fl*^*Pdgfrα*^*CreERT2+*^ and *Tslp*^*fl/fl*^*Nes*^*CreERT2+*^ mice that lack fibroblast TSLP as compared to control mice (Fig. 4c and Extended Data Fig. 9c,d). To further confirm EECs can activate the ILC2–tuft cell circuit through an activated G protein-coupled receptor (GPCR) pathway, we utilized triple-mutant *Vil1*^*Flp*^*Cck*^*Cre*^*R26*^*Dual*−*hM3Dq*^ mice with epithelial lineage-restricted expression of activating designer receptors exclusively activated by designer drugs (DREADDs) on CCK-lineage-restricted intestinal epithelia, which includes preproglucagon^+^ EECs that secrete GLP-2 (ref. ^23^; Fig. 4d and Extended Data Fig. 9e). Following administration of the synthetic ligand clozapine N-oxide (CNO), ILC2s and tuft cells were increased in the small intestine, confirming amplification of the epithelial tuft cell circuit downstream of EEC activation (Fig. 4e–g and Extended Data Fig. 9f,g).

## Discussion

Intestinal telocytes and trophocytes are subepithelial fibroblasts extending from the crypts to the villus tips whose arborizing dendrites establish the critical WNT and BMP counter-gradients that maintain stem cells and control epithelial differentiation during villus transit^15,16,34,35^. Our findings demonstrate that these critical structural cells are the principal intestinal cell sources of TSLP—an alarmin linked with activation of type 2 immune cells, including ILC2s—and are consistent with roles for long-lived structural cells as hubs for tissue immunity^36^. Notably, TSLP expression was prominent in VTTs, specialized *Lgr5*^+^ subepithelial fibroblasts that control epithelial differentiation at villus apices, and positioning them to relay information regarding intestinal content^17^. Further work is needed to assess whether related structural fibroblast subsets in other tissues mediate local TSLP-mediated communication with innate immune cells.

Although activation of ILC2s by food intake was noted^19^, how information regarding luminal content is relayed through epithelia to resident ILC2s remained unclear. EECs sense nutrients and secrete peptide hormones and neurotransmitters that collectively orchestrate intestinal motility, absorption and integration of neuronal circuitry controlling feeding, satiety and positive consumptive or negative aversive reactions^37^. Sophisticated fate-mapping and lineage-marking studies revealed EECs have a longer half-life than enterocytes during villus ascent and are zonated vertically such that peptide hormones are expressed sequentially in overlapping patterns among EEC subsets to optimize small intestinal function^22,23,38^. In our investigation, we verified expression of receptors on telocytes for GLP-2—a proglucagon-derived peptide previously shown to promote release of epithelial growth factors from intestinal subepithelial fibroblasts^39^. Although GLP-2 deletion does not impact small intestine differentiation, it is required for recovery from intestinal atrophy after fasting or injury^40–42^, underpinning the success of stabilized GLP-2 receptor agonists used therapeutically in humans to increase small intestine growth or prevent atrophy following parenteral feeding^27^. Although effects of GLP-2 receptor agonists are reversible after cessation of therapy^43^, the striking intestinotrophic effects of model helminth and protist infections prompted us to query whether this physiologic pathway shared mechanisms with the intestinal response to these organisms, which includes amplification of the ILC2–tuft cell circuit. Whether GLP-1, which is generated in equimolar amounts with GLP-2 after nutrient ingestion, is involved in metabolic adaptations induced by helminth infection and innate type 2 immune pathways will require further study^9,27,28,44^.

Our findings in mouse and human systems show that GLP-2 can stimulate telocytes to produce TSLP, thus linking nutrient ingestion and ILC2-mediated amplification of epithelial tuft cells. Tuft cells are specialized chemosensory cells that express a variety of GPCRs that share signal transduction recognition with type 2 taste receptors and provide feedback amplification of the ILC2–tuft cell circuit by release of a second alarmin, IL-25 (ref. ^45^). As suggested recently, such a system may be part of a regulated food quality control circuit by increasing detection of constituents in ingested cargo that attach positive or negative associations on content that has passed more proximal surveillance mechanisms^46^. Although nutrients stimulate EECs and indirectly activate reversible tuft cell expansion through GLP-2 and TSLP, intestinal protists and helminths can stimulate tuft cell GPCRs directly and sustain the amplified circuit^9,47,48^. By bypassing a physiologic circuit linked with feeding, endemically adapted parasites establish a reproductive niche protected from superinfection by other pathogens while sustaining the host metabolic state^9,49,50^—a condition supported by intestinal lengthening, tuft cell expansion and increased attentiveness to ingested constituents (Extended Data Fig. 9h). Understanding how alarmins like TSLP and IL-25 act combinatorially to regulate innate type 2 immunity in support of local physiologic pathways, as co-opted by intestinal pathobionts to sustain their niche, may enable new strategies for increasing tissue resilience while avoiding the dysregulated state of allergic disease.

## Methods

### Mice

C57BL/6J mice were purchased from the Jackson laboratory (JAX, catalog number 000664) or mutant alleles backcrossed more than eight generations to C57BL/6J. Red5 or B6(C)-*Il5*^*tm1.1(icre)Lky*^/J^19^, Smart13 or B6.129S4(C)-*Il13*^*tm2.1Lky*^/J^10^, *Tslpr*^−*/*−^ (ref. ^51^), backcrossed *Il4ra*^−/−^ or BALB/c-*Il4ra*^*tm1Sz*^/J (JAX, catalog number 003514)^6^, and *Il13ra1*^−/−^ (ref. ^52^ mice generated or obtained by this laboratory have been described. Vil1^Cre^ or B6.Cg-Tg(Vil1-cre)997Gum/J (JAX, catalog number 004586), *Pdgfra*^*CreERT2*^ or B6.129S-*Pdgfra*^*tm1.1(cre/ERT2)Blh*^/J (JAX, catalog number 032770), *Nes*^*CreERT2*^ or C57BL/6-Tg(Nes-cre/ERT2)KEisc/J (JAX, catalog number 016261) were purchased from the Jackson laboratory. C57BL/6 *Pou2f3*^−/−^ (rederived from C57BL/6NTac *Pou2f3*^*tm1.1(KOMP)Vlcg*^) mice^9^ were provided by M. S. Anderson. C57BL/6 *Tslpr*^*fl/fl*^ mice^53^ were provided by S. F. Ziegler. C57BL/6 *Glp2r*^−*/*−^ mice^54^ were provided by D. Drucker. *Lgr5*-eGFP mice^55^ were provided from Genentech by F. de Sauvage and O. D. Klein. Triple-mutant *Vil1*^*Flp*^ *Cck*^*Cre*^ (JAX, catalog number 012706) *R26*^*Dual*−*hM3Dq*^ (JAX, catalog number 026942) mice^23^ were provided by Z. A. Knight.

Flare-TSLP mice were generated by homologous gene targeting in C57BL/6 embryonic stem cells based on a reporter cassette used to target the *Il25* locus^6^. Briefly, a 2.34-kb 3′ homologous arm in the 3′ untranslated region of *Tslp* was amplified from C57BL/6 genomic DNA and a 3.79-kb fragment from exon 1 to the end of coding sequence of exon 5 was amplified to serve as the 5′ homologous arm. A 5′ *loxP* site (target site for Cre recombinase) was inserted 187 bp upstream of exon 4 within the modified 5′ arm. The reporter cassette encoded (5′ to 3′) a *loxP* site (3′*loxP* in final construct), encephalomyocarditis virus internal ribosomal entry site (IRES) element, tandem RFP or tdTomato, bovine growth hormone poly(A) signal and an frt-flanked neomycin selection cassette, and was subcloned between the modified 5′ and 3′ homologous arms with correct orientation using the multiple cloning site of a basal targeting vector, pKO915-DT (Lexicon Genetics). After linearization by *Not*I, the reporter cassette was electroporated into C57BL/6 embryonic stem cells. Following growth on irradiated mouse embryonic fibroblast feeders and selection in G418-containing medium, neomycin-resistant clones were screened for correct 5′ and 3′ homologous recombination by long-range PCR and further confirmed for retention of the 5′*loxP* site. One clone was injected into albino C57BL/6 blastocysts to generate chimeras and males with coats with the highest black-to-white coat color ratio were bred with B6.129S4-*Gt(ROSA)26Sor*^*tm1(FLP1)Dym*^/RainJ females (JAX, catalog number 009086) to excise the neomycin resistance cassette. Offspring with germline transmission and confirmed neo^r^-deleted *Flare-Tslp* allele were backcrossed to C57BL/6 mice to cross out the FLP1 allele. *Flare-Tslp* was genotyped using primers 5′*loxP fw*: 5′-GGAACGAAGTTGAAACACCACGACC-3′ and 5′*loxP rev*: 5’-GGGGATGGGGATAGGAGGGAAAGAC-3′, yielding a 277-bp mutant or a 243-bp WT band. After Cre-*loxP*-mediated deletion, the floxed-out *Tslp* allele can be detected by PCR using primers 5′*loxP fw* (as above) and *IRES rev*: 5′-TTGTTGAATACGCTTGAGGAGAGCC-3′, yielding a 596 base pair band, whereas a nonrecombined floxed band is 2,878 base pairs.

Mice were maintained in the University of California, San Francisco (UCSF) specific pathogen-free animal facility in accordance with the guidelines established by the Institutional Animal Care and Use Committee and Laboratory Animal Resource Center. Unless otherwise specified, mice were kept on 12-h light–dark cycles and ad libitum water and chow (PicoLab, catalog number 5053). The facility was maintained at temperature 68–79 °F and 30–70% humidity. Pooled experiments included female and male mice at 8–12 weeks old. All experimental procedures were approved by the Laboratory Animal Resource Center at the UCSF.

### Antibodies

**Table:** 

Antibodies	Supplier	Catalog number	Dilution
Brilliant violet 421 anti-mouse CD19 antibody	BioLegend	115549	1:200
Brilliant violet 421 anti-mouse TER-119/erythroid cells antibody	BioLegend	116234	1:200
Brilliant violet 421 anti-mouse Ly-6G/Ly-6C (Gr-1) antibody	BioLegend	108445	1:200
Brilliant violet 605 anti-human/mouse/rat CD278 (ICOS) antibody	BioLegend	313538	1:100
Brilliant violet 421 anti-mouse/human CD11b antibody	BioLegend	101251	1:200
Pacific blue anti-mouse CD11c antibody	BioLegend	117322	1:200
Pacific blue anti-mouse CD49b (pan-NK cells) antibody	BioLegend	108918	1:200
Brilliant violet 421 anti-mouse CD335 (NKp46) antibody	BioLegend	137612	1:200
Brilliant violet 421 anti-mouse NK-1.1 antibody	BioLegend	108741	1:200
Brilliant violet 421 anti-mouse TCR γ/δ antibody	BioLegend	118120	1:200
Pacific blue anti-mouse FcεRIα antibody	BioLegend	134314	1:200
Pacific blue anti-mouse F4/80 antibody	BioLegend	123124	1:200
BUV395 rat anti-mouse CD45	BD	564279	1:200
PE/Cyanine7 anti-mouse/human KLRG1 (MAFA) antibody	BioLegend	138416	1:100
APC anti-human CD4 antibody	BioLegend	300514	1:50
Ki-67 monoclonal antibody (SolA15), FITC, eBioscience	Invitrogen	11-5698-82	1:1000
APC anti-mouse CD201 (EPCR) antibody	BioLegend	141506	1:200
PerCP/cyanine5.5 anti-mouse CD31 antibody	BioLegend	102420	1:200
Brilliant violet 421 anti-mouse CD31 antibody	BioLegend	102424	1:200
Brilliant violet 605 anti-mouse CD140α antibody	BioLegend	135916	1:100
Alexa Fluor 488 anti-mouse LYVE1 antibody	eBiosciences	53-0443-82	1:100
Alexa Fluor 488 anti-mouse podoplanin antibody	BioLegend	127406	1:100
BV786 rat anti-mouse CD34	BD	742971	1:100
Alexa Fluor 647 anti-mouse CD326 (EPCAM) antibody	BioLegend	118212	1:100
Alexa Fluor 488 anti-mouse CD326 (EPCAM) antibody	BioLegend	118210	1:100
Alexa Fluor 488 Rat IgG2a, κ isotype Ctrl antibody	BioLegend	400525	1:100
Anti-DCAMKL1 (DCLK1) antibody	Abcam	ab31704	1:500
Mouse podoplanin antibody	R&D	AF3244	1:250
Mouse PDGFR alpha antibody	R&D	MAB1062	1:250
Mouse EPCR (CD201) antibody	R&D	AF2749	1:200
CD34 monoclonal antibody (RAM34), eBioscience	ThermoFisher	14-0341-82	1:500
Anti-green fluorescent protein antibody	Aveslabs	GFP-1020	1:500
Living colors DsRed polyclonal antibody	TakaraBio	632496	1:250
mCherry monoclonal antibody (16D7)	ThermoFisher	M11217	1:500
TSLP monoclonal antibody	Invitrogen	MA5-23779	1:100

### TSLP antibody labeling

Mouse TSLP antibody for flow cytometric analysis was labeled using Alexa Fluor 488 Antibody Labeling Kit (ThermoFisher, catalog number A20181) following the manufacturerʼs protocol.

### Flow cytometry and FACS

Small intestinal tissues were harvested at indicated times and single-cell suspensions of epithelium or lamina propria were obtained. In brief, 6-cm lengths of proximal jejunum or distal ileum were collected and flushed with ice-cold PBS. After removing the Peyer’s patches, gut tissues were opened and washed with PBS to remove lumen content and mucus. Segments were incubated while rocking for 15 min at 37 °C in 20 ml HBSS (Ca^2+^-free and Mg^2+^-free) containing 5% FCS, 10 mM HEPES (Sigma, catalog number H3537), 10 mM EDTA (Corning, catalog number 46-034-CI) and 10 mM dithiothreitol (DTT; Sigma, catalog number D0632). Tissue segments were vortexed vigorously for 30 s and supernatants collected and centrifuged. The pelleted epithelial fraction was washed once with PBS before staining for flow cytometry. The remaining tissue segments were incubated a second time while rocking for 15 min at 37 °C in 10 ml of the same HBSS/FBS/EDTA/DTT medium and then vortexed for 30 s. The tissue segments were transferred to new tubes and incubated while rocking for 5 min at 37 °C in 20 ml HBSS (with Ca^2+^ and Mg^2+^) containing 2% FCS and 10 mM HEPES before transferring to new tubes containing 5 ml HBSS (with Ca^2+^ and Mg^2+^), 3% FCS, 10 mM HEPES, 30 µg ml^−1^ DNase (Roche, catalog number 10104159001) and 0.1 U ml^−1^ Liberase TM (Roche, catalog number 5401127001) in C tubes (Miltenyi Biotec, catalog number 130-096-334). Samples were processed with gentleMax Octo Dissociator program ‘intestine.’ Lamina propria cells were pelleted, washed and filtered with 70 µM cell strainers before staining for flow cytometry. Standard surface and/or intracellular staining protocols were used followed by 4′,6-diamidino-2-phenylindole (DAPI) or Live/Dead Viability dye (ThermoFisher) staining for dead cell exclusion. Samples were processed using a BD LSRFortessa (Bectin Dickinson) flow cytometer and analyzed using FlowJo software. Cell sorting experiments were performed on MoFlo (Beckman Coulter) or BD FACSAria.

### Primary mouse fibroblast culture

siLP cells were isolated as above. Cells were suspended in fibroblast culture media (DEME/F12 supplement with 10% FBS and penicillin/streptomycin) and incubated at 37 °C with 5% CO_2_ for 4 h to allow fibroblast adherence before washing off nonadherent cells. The remaining adherent cells were incubated in fresh culture medium. Cells were plated at 1 × 10^5^ cells per well in 48-well plates or 2 × 10^5^ cells per well in 24-well plates and incubated overnight before use.

### Human intestinal fibroblast cell lines

#### Study approval

The study was conducted in accordance with the principles of the Declaration of Helsinki and was approved by the Institutional Review Board of the University of California, San Francisco (19-27302). All participants provided written informed consent before inclusion.

### Study participants and biospecimen collection

Patients underwent colonoscopy or sigmoidoscopy for noninflammatory indications (for example, colorectal cancer screening) and were referred as healthy controls (HC). Baseline demographic information for the study participants are provided in Supplementary Table 1. Patients consented to publish deidentified patient demographics including age at the time of sample collection, sex, diagnosis and medical center. Demographic options were defined by the investigators and participants chose their classifications.

### Sample collection and storage

Biopsies from colon and ileum were obtained with standard cold endoscopic biopsy forceps and collected in a conical tube with Basal Media (Advanced DMEM/F12 with nonessential amino acids and sodium pyruvate (Thermo, catalog number 12634-010), 2 mM Glutamax (Thermo, catalog number 35050061), 10 mM HEPES (Corning), penicillin-streptomycin-neomycin (PSN) Antibiotic Mixture (Thermo, catalog number 15640055), 100 µg ml^−1^ Normocin (Invivogen, catalog number ant-nr-2), 1 mM N-acetylcysteine (Sigma-Aldrich, catalog number A9165)) with 10 µM Y-27632 (MedChem Express) at 4 °C. Samples were immediately placed on ice and transported to the laboratory for processing as described^56^. Biopsies were transferred into cryovials containing freezing medium (90% (v/v) FCS, 10% (v/v) dimethylsulfoxide (DMSO) and 10 µM Y-27632) and placed immediately into a freezing container (Coolcell) and stored at −70 °C for up to 4 weeks before transferring to liquid nitrogen cryostorage until further processing.

### Establishment of patient-derived intestinal fibroblast cell lines

Biopsies were thawed for 2.5 min with gentle agitation in a 37 °C water bath, transferred to a 50-ml tube, washed twice with basal medium containing 10 µM Y-27632, and then incubated in 2 ml digestion buffer (basal medium with 10 µM Y-27632, 600 U ml^−1^ Collagenase IV (Worthington, catalog number LS004189), 0.1 mg ml^−1^ DNAse I (Sigma-Aldrich, catalog number D4513)). Biopsies were digested for 20 min at 37 °C in a shaking incubator set at 225 rpm. Samples were pipetted vigorously after incubation. The suspension was passed through a 100-µm strainer (pluriSelect, catalog number 43-100100-40) over a 5-ml tube, and the filter was washed twice with HBSS (Corning), containing 0.1 mg ml^−1^ DNAse I. The suspension was centrifuged at 450*g* for 5 min at 4 °C. One additional wash was performed in HBSS containing 0.1 mg ml^−1^ DNAse I and centrifuged at 450*g* for 5 min at 4 °C. The pellet was resuspended in prewarmed human fibroblast culture medium (EMEM-Eagle’s minimal essential medium with l-glutamine (Quality Biological, catalog number 112-018-101), PSN, 100 µg ml^−1^ Normocin (InvivoGen, catalog number ant-nr-2), FCS 20% (v/v) and 50 ng ml^−1^ recombinant human FGF-basic (PeproTech, catalog number 100-18B)] and plated in a 24-well plate. The culture medium was replaced after 24 h in culture. After expansion, fibroblast lines were transferred to T25 flasks and passaged twice a week at a ratio of 1:3. Aliquots of low-passage (<8) patient-derived intestinal fibroblasts were cryopreserved in 90% FCS with 10% DMSO.

Cells used in this paper were thawed from frozen stocks, expanded in human fibroblast culture medium and maintained in cell incubator at 37 °C with 5% CO_2_. Cells were seeded at 2 × 10^5^ cells per well in 24-well plates and incubated overnight before use.

### TSLP ELISA from mouse intestinal homogenates and explants

Samples from proximal jejunum (2 cm) or distal ileum (3 cm) were weighed and harvested in RIPA (ThermoFisher, catalog number 89900) buffer containing proteinase inhibitors in M tubes (Miltenyi Biotec, catalog number 130-096-335) followed by dissociation with gentleMax Octo Dissociator program ‘protein 1.’ Supernatants from the homogenates were collected following brief centrifugation and stored at −80 °C. Intestinal explants were cultured in 96-well plates with complete RPMI 1640 medium supplemented with 10% FBS for 5 h at 37 °C in a 5% CO_2_ incubator. After incubation, supernatants were collected following brief centrifugation and stored at −80 °C. TSLP ELISA was performed (Biolegend Deluxe TSLP ELISA kit; BioLegend, catalog number 434104) with undiluted homogenates or tissue explant culture supernatants. Protein concentrations were normalized by tissue weight of the starting samples.

### Drug treatments

MC903 (Calcipotriol, Tocris Biosciences, catalog number 2700; 100 µM working concentration in ethanol) was applied topically on shaved back skin using 60 µl per mouse daily for 7 consecutive days^14^. Stabilized human GLP-2[Gly2] (Teduglutide, Echelon Bioscience, catalog number 471-21) was given at 10 µg per mouse subcutaneously (s.c.) in 100 µl DMSO–PBS solution consecutively for 3 days or as indicated at 25 µg per mouse intraperitoneally (i.p.) 2 h before collecting tissues. Tamoxifen (Sigma, catalog number T5648) was administrated at 3 mg per mouse or 100 mg kg^−1^ in 100 µl corn oil i.p. daily for 7 days; mice were rested for 3–5 days before experimental use. Alternatively, tamoxifen diet (Inotive, catalog number TD.130858) was given to mice ad libitum for at least 4 weeks before experimental use. CNO (Cayman, catalog number 16882) was given 5 mg kg^−1^ i.p. for 3 consecutive days. EdU (Sigma, catalog number 900584) was given i.p. at 1 mg per mouse in 100 µl PBS 24 h before tissue harvests.

For signaling pathway analysis, mouse fibroblasts, mouse intestinal explants and human fibroblasts were treated with agonists including GLP-2[Gly2] 500 nM and forskolin 100 µM (Tocris, catalog number 1099), or inhibitors including 666-15 10 µM (Tocris, catalog number 5661), LY294002 75 µM (Tocris, catalog number 1130), rapamycin 2.5 µM (Biogems, catalog number 1672186) and PD98059 50 µM (TCI, catalog number R0097) in 200 µl of culture medium. Inhibitors were added 1 h before agonists. For TSLP measurement by ELISA (BioLegend, catalog number 521904 for human TSLP; BioLegend, catalog number 434104 for mouse TSLP), supernatants were collected 5 h after agonist stimulation. For TSLP measurement by intracellular staining and flow cytometric analysis, cells were incubated for 4 h with Brefeldin A (BioLegend, catalog number 42601) given 1 h after agonists before cells were harvested and analyzed using standard cell surface and intracellular staining.

### *N. brasiliensis* and *T. muris* infection

*N.* *brasiliensis* was maintained in rats and purified as described^6^. Mice were injected s.c. with 500 purified L3 larvae and small intestines were collected on days 5, 7 and 12 after infection for analysis of tuft cells as described^6^.

Two hundred embryonated *T.* *muris* eggs were orally gavaged per mouse. Cecum and large intestines were analyzed 21 days later.

### Fasting–feeding protocols

For measuring small intestinal TSLP, mice were fasted 16 h overnight before oral gavage with 500 µl food slurry (modified powdered diet formula TekladTD.88232 with comparable nutrient distribution as standard chow diet) in water containing 1.965 kcal for ~1% daily caloric intake) or water as a volumetric control. For small intestinal ILC2 activation, mice were fasted overnight for 16 h before restoring access to standard chow diet (PicoLab, catalog number 5058) and water ad libitum or maintaining on water. Tissues were harvested at indicated timepoints. Overnight fasting followed by water gavage or water access ad libitum was designated the ‘Fasted’ control group; overnight fasting followed by food gavage or food access ad libitum was designated the ‘Fasted+refedʼ group.

### Tissue immunofluorescence

Tissues were fixed in 4% paraformaldehyde (PFA; Electron, catalog number 15710) for 2 h at room temperature or longer at 4 °C, washed three times in PBS and incubated in 30% (w/v) sucrose-PBS solution overnight at 4 °C. Tissues were embedded in optimal cutting temperature compound (OCT; VWR, catalog number 25608-930) on dry ice and stored at −80 °C. Skin samples were cut into 1 cm × 2 cm pieces before fixation. Samples from the small intestine (~10 cm) or proximal large intestine were cut and flushed with PBS before fixation and coiled into ‘Swiss rolls’ before embedding. OCT-embedded tissues were cut into 8–14-µm regular sections or 100-µm thick sections using a Cryostat (Leica, catalog number CM3050 S) and stored at −80 °C. Sections were incubated with 0.02% Triton X-100 (Sigma, catalog number X100) in TNB buffer (Tris-NaCl blocking buffer: 0.1 M Tris-HCl, pH 7.5; 0.15 M NaCl; blocking reagent, Akoya Biosciences, catalog number FP1020) for permeabilization followed by blocking with 5% serum matching the source of the secondary antibodies for 30 min at room temperature. After a 2-h incubation with primary antibodies at room temperature, sections were washed three times in PBS and incubated with secondary antibodies for 1 h at room temperature. Sections were incubated with DAPI for 5 min and washed three times in PBS before mounting. EdU staining was performed using Click-IT Plus EdU imaging kit (Invitrogen, catalog number C10637) following manufacture protocol. Stained samples were visualized using a Nikon A1R confocal microscope with a ×20 objective and analyzed using NIS Element and ImageJ software. Thick section staining protocols were as described^57^ and imaging was processed using Imaris software three-dimensional analysis workstation for Cell Biology Research (Oxford Instruments).

### Tuft cell quantifications

For tuft cell quantification, images were acquired using the ×20 objective and total doublecortin-like kinase 1 (DCLK1)^+^EPCAM^+^ cells in each field were counted and normalized by total number of villus–crypt axes in the field.

### qPCR and gene expression analysis

For analysis of designated populations, cells were purified by FACS directly into lysis buffer followed by RNA extraction using a Qiagen kit (catalog number 74034). cDNA was synthesized using Invitrogen SuperScript IV VILO kit (Invitrogen, catalog number 11754250). qPCR reactions were performed using Taqman probes (Invitrogen) and Taqman gene expression master mix (Invitrogen, catalog number 4369016). Transcripts were normalized to 18S expression. Probe information is as follows:

**Table:** 

Gene	Probe
*Rspo3*	Mm00661105_m1
*Lgr5*	Mm00438890_m1
*Foxl1*	Mm00514937_s1
*Bmp4*	Mm00432087_m1
*Wnt2b*	Mm00437330_m1
*Wnt5a*	Mm00437347_m1
*Grem1*	Mm00488615_s1
*Bmp7*	Mm00432102_m1
*18S*	Mm03928990_g1
*Tslp*	Mm00498739_m1
*Nes*	Mm00450205_m1

### scRNA-seq and cell hashing

Live tdTomato^+^ cells were purified using FACS and labeled with unique barcoded H-2/CD45 TotalSeq-B anti-mouse hashtag antibodies (Biolegend catalog numbers 155831, 155833, 155835, 155837, 155839, 155841) for multiplexing. The cells were processed according to the manufacturer’s instructions using the Chromium Next Gel Beads-in-Emulsion (GEM) Single Cell 3′ Kit v.3.1 (10x Genomics, catalog number 1000269). Briefly, cell counts were performed using a hemocytometer and the desired number of cells was injected into microfluidic chips (10x Genomics, catalog number 1000127) to create GEMs with the 10x Chromium controller. RT was performed on the GEMs followed by purification and amplification of the RT products. Cell multiplexing oligo (CMO) DNA was separated from cDNA using size selection with SPRI select beads (Beckman Coulter, catalog number B23318). Gene expression libraries and CMO libraries were generated separately and profiled using the Bioanalyzer High Sensitivity DNA kit (Agilent Technologies, catalog number 5067-4626). Before sequencing, the gene expression and corresponding CMO libraries were mixed in a 4:1 ratio. Sequencing of the mixed libraries was conducted on the Illumina NovaSeq X platform.

### scRNA-seq data analysis

Sample demultiplexing, alignments and unique molecular identifier counts were performed using the 10x Genomics CellRanger pipeline (v.8.1.0) based on the latest mouse genome (mm39) with a custom reference to include the tdTomato sequence. Doublets were removed using the DoubletFinder (v.2.0.4) algorithm and ambient RNA was eliminated with the SoupX algorithm (v.1.6.2) using default parameters. After preprocessing datasets were analyzed using the Seurat single-cell analysis pipeline (v.5.0.3) in R (v.4.3.1). Quality control involved excluding low-quality cells based on the following criteria: nFeature_RNA between 1,000 and 7,500, nCount_RNA between 2,500 and 40,000 and mitochondrial contamination below 10%. Following filtering, data were normalized using the NormalizeData function with the ‘LogNormalize’ method and the 2,000 most variable genes were identified and scaled before performing principal component analysis. Thirty principal components were used for graph-based clustering (resolution = 0.5) followed by uniform manifold approximation and projection dimensionality reduction.

An additional filtering step based on *tdTomato* expression removed contaminant clusters with fewer than 0.01% *tdTomato*^*+*^ cells and fewer than five absolute *tdTomato*^*+*^ cells. Initial cell type annotation was conducted automatically using scType^58^ followed by manual refinement. Differential gene expression (DE) analysis between cell types in the small intestine samples were conducted using Wilcoxon rank sum tests as implemented in the Seurat function FindAllMarkers. For DE anlaysis between tissues, counts from each sample were summed and followed by pseudobulk DE analysis using limma-voom.

### Public scRNA-seq data analysis

Mouse^15,16^ and human^24,25^ datasets (raw or preprocessed data) were obtained from the single-cell portal of the Broad Institute (https://singlecell.broadinstitute.org). Data were reanalyzed and plotted using Seurat package in the R program.

### Statistics

Quantitative data were represented as mean ± s.e.m. of at least duplicate biological replicates. Studentʼs *t*-test was used for analyses of difference between two groups. Analysis of variance (ANOVA) was used for analyzing experiments with more than two groups adjusted for multiple comparisons. Significance level was set at *α* = 0.05. Statistical analysis was performed using Prism (GraphPad Software). *P < 0.05, **P < 0.01, ***P < 0.001, ****P < 0.0001.

### Reporting summary

Further information on research design is available in the Nature Portfolio Reporting Summary linked to this article.

## Online content

Any methods, additional references, Nature Portfolio reporting summaries, source data, extended data, supplementary information, acknowledgements, peer review information; details of author contributions and competing interests; and statements of data and code availability are available at 10.1038/s41590-025-02328-y.

## Supplementary information

Supplementary Information Supplementary Table 1.

Reporting Summary

Supplementary Videos 1–7 Videos 1–7. Three-dimensional visualization of small intestine. Immunofluorescent staining of jejunum (Videos 1–4) and ileum (Videos 5–7) from Flare-TSLP mice. Red, TSLP-tdTomato; green, PDGFRa; white, EPCAM; blue, DAPI; ×20 objective.

## Data Availability

All raw and processed scRNA-seq data generated in this study have been deposited in the NCBI Gene Expression Omnibus (GEO) under accession number GSE280635. All the other data supporting the findings of this study are available upon request.

## Code availability

This study did not generate new algorithm or code. The custom code and scripts used for analyses and generating related graphs are available upon requests.

## References

[1] Godinho-Silva, C. et al. Light-entrained and brain-tuned circadian circuits regulate ILC3s and gut homeostasis. *Nature* **574**, 254–258 (2019). 31534216 10.1038/s41586-019-1579-3 PMC6788927

[2] Sullivan, Z. A. et al. γδ T cells regulate the intestinal response to nutrient sensing. *Science* **371**, eaba8310 (2021). 33737460 10.1126/science.aba8310 PMC11617329

[3] Lyu, M. et al. ILC3s select microbiota-specific regulatory T cells to establish tolerance in the gut. *Nature* **610**, 744–751 (2022). 36071169 10.1038/s41586-022-05141-x PMC9613541

[4] Jarade, A. et al. Inflammation triggers ILC3 patrolling of the intestinal barrier. *Nat. Immunol.* **23**, 1317–1323 (2022). 35999393 10.1038/s41590-022-01284-1 PMC9477741

[5] Ou, R. & Murphy, K. M. What’s in a name: clarifying the identify of RORgt^+^ antigen-presenting cells. *J. Exp. Med.* **222**, e20250760 (2025). 40372310 10.1084/jem.20250760 PMC12080405

[6] von Moltke, J., Ji, M., Liang, H.-E. & Locksley, R. M. Tuft-cell-derived IL-25 regulates an intestinal ILC2-epithelial response circuit. *Nature* **529**, 221–225 (2016). 26675736 10.1038/nature16161 PMC4830391

[7] Gerbe, F. et al. Intestinal epithelial tuft cells initiate type 2 mucosal immunity to helminth parasites. *Nature* **529**, 226–230 (2016). 26762460 10.1038/nature16527 PMC7614903

[8] Howitt, M. R. et al. Tuft cells, taste-chemosensory cells, orchestrate parasite type 2 immunity in the gut. *Science* **351**, 1329–1333 (2016). 26847546 10.1126/science.aaf1648 PMC5528851

[9] Schneider, C. et al. A metabolite-triggered tuft cell-ILC2 circuit drives small intestinal remodeling. *Cell* **174**, 271–284 (2018). 29887373 10.1016/j.cell.2018.05.014 PMC6046262

[10] Van Dyken, S. J. et al. A tissue checkpoint regulates type 2 immunity. *Nat. Immunol.* **17**, 1381–1387 (2016). 27749840 10.1038/ni.3582 PMC5275767

[11] Vannella, K. M. et al. Combinatorial targeting of TSLP, IL-25, and IL-33 in type 2 cytokine-driven inflammation and fibrosis. *Sci. Transl. Med.* **8**, 337ra65 (2016). 27147589 10.1126/scitranslmed.aaf1938

[12] Taylor, B. C. et al. TSLP regulates intestinal immunity and inflammation in mouse models of helminth infection and colitis. *J. Exp. Med.* **206**, 655–667 (2009). 19273626 10.1084/jem.20081499 PMC2699121

[13] Haber, A. L. et al. A single-cell survey of the small intestinal epithelium. *Nature* **551**, 333–339 (2017). 29144463 10.1038/nature24489 PMC6022292

[14] Li, M. et al. Topical vitamin D3 and low-calcemic analogs induce thymic stromal lymphopoietin in mouse keratinocytes and trigger an atopic dermatitis. *Proc. Natl Acad. Sci. USA* **103**, 11736–11741 (2006). 16880407 10.1073/pnas.0604575103 PMC1544239

[15] Shoshkes-Carmel, M. et al. Subepithelial telocytes are an important source of Wnts that supports intestinal crypts. *Nature* **557**, 242–246 (2018). 29720649 10.1038/s41586-018-0084-4 PMC5966331

[16] McCarthy, N. et al. Distinct mesenchymal cell populations generate the essential intestinal BMP signaling gradient. *Cell Stem Cell* **26**, 391–402.e5 (2020). 32084389 10.1016/j.stem.2020.01.008 PMC7412576

[17] Bahar Halpern, K. et al. Lgr5+ telocytes are a signaling source at the intestinal villus tip. *Nat. Commun.* **11**, 1936 (2020). 32321913 10.1038/s41467-020-15714-x PMC7176679

[18] Melissari, M.-T. et al. Col6a1^+^CD201^+^ mesenchymal cells regulate intestinal morphogenesis and homeostasis. *Cell. Mol. Life Sci.* **79**, 1 (2021). 34910257 10.1007/s00018-021-04071-7 PMC11073078

[19] Nussbaum, J. C. et al. Type 2 innate lymphoid cells control eosinophil homeostasis. *Nature* **502**, 245–248 (2013). 24037376 10.1038/nature12526 PMC3795960

[20] Ricardo-Gonzalez, R. R. et al. Tissue-specific pathways extrude activated ILC2s to disseminate type 2 immunity. *J. Exp. Med.* **217**, e20191172 (2020). 32031571 10.1084/jem.20191172 PMC7144525

[21] Buechler, M. B. et al. Cross-tissue organization of the fibroblast lineage. *Nature* **593**, 575–579 (2021). 33981032 10.1038/s41586-021-03549-5

[22] Gehart, H. et al. Identification of enteroendocrine regulators by real-time single-cell differentiation mapping. *Cell* **176**, 1158–1173 (2019). 30712869 10.1016/j.cell.2018.12.029

[23] Bai, L. et al. Enteroendocrine cell types that drive food reward and aversion. *eLife* **11**, e74964 (2022). 35913117 10.7554/eLife.74964 PMC9363118

[24] Smillie, C. S. et al. Intra- and inter-cellular rewiring of the human colon during ulcerative colitis. *Cell* **178**, 714–730 (2019). 31348891 10.1016/j.cell.2019.06.029 PMC6662628

[25] Korsunsky, I. et al. Cross-tissue, single-cell stromal atlas identifies shared pathological fibroblast phenotypes in four chronic inflammatory diseases. *Med* **3**, 481–518 (2022). 35649411 10.1016/j.medj.2022.05.002 PMC9271637

[26] Brubaker, P. L. Glucagon-like peptide-2 and the regulation of intestinal growth and function. *Compr. Physiol.* **8**, 1185–1210 (2018). 29978894 10.1002/cphy.c170055

[27] O’Rahilly, S. The islet’s bridesmaid becomes the bride: proglucagon-derived peptides deliver transformative therapies. *Cell* **184**, 1945–1948 (2021). 33831374 10.1016/j.cell.2021.03.019

[28] Drucker, D. J. Mechanisms of action and therapeutic application of glucagon-like peptide-1. *Cell Metab.* **27**, 740–756 (2018). 29617641 10.1016/j.cmet.2018.03.001

[29] de Heuvel, E., Wallace, L., Sharkey, K. A. & Sigalet, D. L. Glucagon-like peptide 2 induces vasoactive intestinal polypeptide expression in enteric neurons via phophatidylinositol 3-kinase-g signaling. *Am. J. Physiol. Endocrinol. Metab.* **303**, E9941005 (2012). 10.1152/ajpendo.00291.2012 PMC3469609 22895780

[30] Yusta, B. et al. Identification of glucagon-like peptide-2 (GLP-2)-activated signaling pathways in baby hamster kidney fibroblasts expressing the rat GLP-2 receptor. *J. Biol. Chem.* **274**, 30459–30467 (1999). 10521425 10.1074/jbc.274.43.30459

[31] Koehler, J. A., Harper, W., Barnard, M., Yusta, B. & Drucker, D. J. Glucagon-like peptide-2 does not modify the growth or survival of murine or human intestinal tumor cells. *Cancer Res.* **68**, 7897–7904 (2008). 18829546 10.1158/0008-5472.CAN-08-0029 PMC3606135

[32] Futamura, K. et al. beta2-Adrenoceptor agonists enhance cytokine-induced release of thymic stromal lymphopoietin by lung tissue cells. *Int. Arch. Allergy Immunol.* **152**, 353–361 (2010). 20185927 10.1159/000288288

[33] Ma, L., Zhen, J. & Sorisky, A. Regulators of thymic stromal lymphopoietin production by human adipocytes. *Cytokine* **136**, 155284 (2020). 32950025 10.1016/j.cyto.2020.155284

[34] Kondo, A. & Kaestner, K. H. Emerging diverse roles of telocytes. *Development* **146**, dev175018 (2019). 31311805 10.1242/dev.175018 PMC6679369

[35] Beumer, J. et al. BMP gradient along the intestinal villus axis controls zonated enterocyte and goblet cell states. *Cell Rep.* **38**, 110438 (2022). 35235783 10.1016/j.celrep.2022.110438

[36] Krausgruber, T. et al. Structural cells are key regulators of organ-specific immune responses. *Nature* **583**, 296–302 (2020). 32612232 10.1038/s41586-020-2424-4 PMC7610345

[37] Gribble, F. M. & Reimann, F. Enteroendocrine cells: chemosensors in the intestinal epithelium. *Annu. Rev. Physiol.* **78**, 277–299 (2016). 26442437 10.1146/annurev-physiol-021115-105439

[38] Beumer, J. et al. Enteroendocrine cells switch hormone expression along the crypt-to-villus BMP signalling gradient. *Nat. Cell Biol.* **20**, 909–916 (2018). 30038251 10.1038/s41556-018-0143-y PMC6276989

[39] Leen, J. L. S. et al. Mechanism of action of glucagon-like peptide-2 to increase IGF-I mRNA in intestinal subepithelial fibroblasts. *Endocrinology* **152**, 436–446 (2011). 21159855 10.1210/en.2010-0822 PMC3384785

[40] Wismann, P. et al. The endogenous preproglucagon system is not essential for gut growth homeostasis in mice. *Mol. Metab.* **6**, 681–692 (2017). 28702324 10.1016/j.molmet.2017.04.007 PMC5485241

[41] Shin, E. D., Estall, J. L., Izzo, A., Drucker, D. J. & Brubaker, P. L. Mucosal adaptation to enteral nutrients is dependent on the physiologic actions of glucagon-like peptide-2 in mice. *Gastroenterology* **128**, 1340–1353 (2005). 15887116 10.1053/j.gastro.2005.02.033

[42] Nelson, D. W. et al. Insulin-like growth factor I and glucagon-like peptide-2 responses to fasting followed by controlled or ad libitum refeeding in rats. *Am. J. Physiol. Regul. Integr. Comp. Physiol.* **294**, R1175–R1184 (2008). 18256135 10.1152/ajpregu.00238.2007

[43] Tsai, C. H., Hill, M., Asa, S. L., Brubaker, P. L. & Drucker, D. J. Intestinal growth-promoting properties of glucagon-like peptide-2 in mice. *Am. J. Physiol.* **273**, E77–E84 (1997). 9252482 10.1152/ajpendo.1997.273.1.E77

[44] Rankin, L. C. & Artis, D. Beyond host defense: emerging functions of the immune system in regulating complex tissue physiology. *Cell* **173**, 554–567 (2018). 29677509 10.1016/j.cell.2018.03.013

[45] Schneider, C., O’Leary, C. E. & Locksley, R. M. Regulation of immune responses by tuft cells. *Nat. Rev. Immunol.* **19**, 584–593 (2019). 31114038 10.1038/s41577-019-0176-x PMC8331098

[46] Florsheim, E. B., Sullivan, Z. A., Khoury-Hanold, W. & Medzhitov, R. Food allergy as a biological food quality control system. *Cell* **184**, 1440–1454 (2021). 33450204 10.1016/j.cell.2020.12.007

[47] Nadjsombati, M. S. et al. Detection of succinate by intestinal tuft cells triggers a type 2 innate immune circuit. *Immunity* **49**, 33–41 (2018). 30021144 10.1016/j.immuni.2018.06.016 PMC6084797

[48] Luo, X.-C. et al. Infection by the parasitic helminth *Trichinella spiralis* activates a Tas2r-mediated signaling pathway in intestinal tuft cells. *Proc. Natl Acad. Sci. USA* **116**, 5564–5569 (2019). 30819885 10.1073/pnas.1812901116 PMC6431192

[49] Chudnovskiy, A. et al. Host–protozoan interactions protect from mucosal infections through activation of the inflammasome. *Cell* **167**, 444–456 (2016). 27716507 10.1016/j.cell.2016.08.076 PMC5129837

[50] Ramanan, D. et al. Helminth infection promotes colonization resistance via type 2 immunity. *Science* **352**, 608–612 (2016). 27080105 10.1126/science.aaf3229 PMC4905769

[51] Carpino, N. et al. Absence of an essential role for thymic stromal lymphopoietin receptor in murine B-cell development. *Mol. Cell. Biol.* **24**, 2584–2592 (2004). 14993294 10.1128/MCB.24.6.2584-2592.2004 PMC355866

[52] Ricardo-Gonzalez, R. R. et al. Innate type 2 immunity controls hair follicle commensalism by *Demodex* mites. *Immunity* **55**, 1891–1908 (2022). 36044899 10.1016/j.immuni.2022.08.001 PMC9561030

[53] Han, H., Thelen, T. D., Comeau, M. R. & Ziegler, S. F. Thymic stromal lymphopoietin-mediated epicutaneous inflammation promotes acute diarrhea and anaphylaxis. *J. Clin. Invest.* **124**, 5442–5452 (2014). 25365222 10.1172/JCI77798 PMC4348967

[54] Yusta, B. et al. ErbB signaling is required for the proliferative actions of GLP-2 in the murine gut. *Gastroenterology* **137**, 986–996 (2009). 19523469 10.1053/j.gastro.2009.05.057

[55] Tian, H. et al. A reserve stem cell population in small intestine renders Lgr5-positive cells dispensable. *Nature* **478**, 255–259 (2011). 21927002 10.1038/nature10408 PMC4251967

[56] Mennillo, E. et al. Single-cell and spatial multi-omics highlight effects of anti-integrin therapy across cellular compartments in ulcerative colitis. *Nat. Commun.* **15**, 1493 (2024). 38374043 10.1038/s41467-024-45665-6 PMC10876948

[57] Li, W., Germain, R. N. & Gerner, M. Y. High-dimensional cell-level analysis of tissues with Ce3D multiplex volume imaging. *Nat. Protoc.* **14**, 1708–1733 (2019). 31028373 10.1038/s41596-019-0156-4 PMC8690297

[58] Ianevski, A., Giri, A. K. & Aittokallio, T. Fully-automated and ultra-fast cell-type identification using specific marker combinations from single-cell transcriptomic data. *Nat. Commun.* **13**, 1246 (2022). 35273156 10.1038/s41467-022-28803-w PMC8913782

[59] Small intestine from C57BL/6 mice (v1, 150×150), Visium HD Spatial Gene Expression Library by Space Ranger v3.0. *10x Genomics* https://www.10xgenomics.com/datasets/visium-hd-cytassist-gene-expression-libraries-of-mouse-intestine?noTrack=true (2024).

