# Supplementary material for: TSLP links intestinal nutrient sensing with amplification of the ILC2–tuft cell circuit
Source: Nat Immunol. 2025 Nov 12;26(12):2218–26. doi: 10.1038/s41590-025-02328-y (PMC12643938; doi:10.1038/s41590-025-02328-y)
Supplement: Supplementary file 1 — Supplementary Table 1. [file 41590_2025_2328_MOESM1_ESM.pdf]

nature immunology

Supplementary information https://doi.org/10.1038/s41590-025-02328-y

TSLP links intestinal nutrient sensing with amplification of the ILC2–tuft cell circuit

In the format provided by the authors and unedited

Supplementary Table S1. Baseline demographic information for study participants.

Patient ID	Age (y)	Sex	Disease status	Tissue
Subject1	40-49	M	HC	colon
Subject2	40-49	M	HC	colon
Subject3	50-59	F	HC	ileum
