# Supplementary material for: TSLP links intestinal nutrient sensing with amplification of the ILC2–tuft cell circuit
Source: Nat Immunol. 2025 Nov 12;26(12):2218–26. doi: 10.1038/s41590-025-02328-y (PMC12643938; doi:10.1038/s41590-025-02328-y)
Supplement: Supplementary file 2 — Reporting Summary [file 41590_2025_2328_MOESM2_ESM.pdf]

nature portfolio

Corresponding author(s): Richard M. Locksley

Last updated by author(s): Sept 26, 2025

Reporting Summary

Nature Portfolio wishes to improve the reproducibility of the work that we publish. This form provides structure for consistency and transparency in reporting. For further information on Nature Portfolio policies, see our Editorial Policies and the Editorial Policy Checklist.

Please do not complete any field with "not applicable" or n/a. Refer to the help text for what text to use if an item is not relevant to your study.
For final submission: please carefully check your responses for accuracy; you will not be able to make changes later.

Statistics

For all statistical analyses, confirm that the following items are present in the figure legend, table legend, main text, or Methods section.

n/a	Confirmed	
[ ]	[x]	The exact sample size (*n*) for each experimental group/condition, given as a discrete number and unit of measurement
[ ]	[x]	A statement on whether measurements were taken from distinct samples or whether the same sample was measured repeatedly
[ ]	[x]	The statistical test(s) used AND whether they are one- or two-sided<br>*Only common tests should be described solely by name; describe more complex techniques in the Methods section.*
[x]	[ ]	A description of all covariates tested
[ ]	[x]	A description of any assumptions or corrections, such as tests of normality and adjustment for multiple comparisons
[ ]	[x]	A full description of the statistical parameters including central tendency (e.g. means) or other basic estimates (e.g. regression coefficient) AND variation (e.g. standard deviation) or associated estimates of uncertainty (e.g. confidence intervals)
[ ]	[x]	For null hypothesis testing, the test statistic (e.g. *F*, *t*, *r*) with confidence intervals, effect sizes, degrees of freedom and *P* value noted<br>*Give P values as exact values whenever suitable.*
[x]	[ ]	For Bayesian analysis, information on the choice of priors and Markov chain Monte Carlo settings
[x]	[ ]	For hierarchical and complex designs, identification of the appropriate level for tests and full reporting of outcomes
[x]	[ ]	Estimates of effect sizes (e.g. Cohen's *d*, Pearson's *r*), indicating how they were calculated

Our web collection on statistics for biologists contains articles on many of the points above.

Software and code

Policy information about availability of computer code

Data collection

Single cell RNA sequencing (sc-RNAseq) and cell hashing
Live tdTomato+ cells were purified using FACS and labeled with unique barcoded H-2/CD45 TotalSeqTM-B anti-mouse hashtag antibodies (Biolegend Cat. 155831, 155833, 155835, 155837, 155839, 155841) for multiplexing. The cells were processed according to the manufacturer's instructions using the ChromiumTM Next GEM Single Cell [illegible]’ Kit v3.1 (10X Genomics, Cat. 1000269). Briefly, cell counts were performed using a hemacytometer and the desired number of cells was injected into microfluidic chips (10X Genomics, Cat. 1000127) to create Gel Beads-in-Emulsion (GEMs) with the 10X Chromium controller. Reverse transcription (RT) was performed on the GEMs followed by purification and amplification of the RT products. Cell multiplexing oligo (CMO) DNA was separated from cDNA using size selection with SPRI select beads (Beckman Coulter, Cat. B23318). Gene expression (GEX) libraries and CMO libraries were generated separately and profiled using the Bioanalyzer High Sensitivity DNA kit (Agilent Technologies, Cat. 5067-4626). Prior to sequencing, the GEX and corresponding CMO libraries were mixed in a 4:1 ratio. Sequencing of the mixed libraries was conducted on the Illumina NovaSeq X platform.

Data analysis

sc-RNAseq data analysis
Sample demultiplexing, alignments and UMI counts were performed using the 10x Genomics CellRanger pipeline (v 8.1.0) based on the latest mouse genome (mm39) with a custom reference to include the tdTomato sequence. Doublets were removed using the DoubletFinder (v2.0.4) algorithm and ambient RNA was eliminated with the SoupX algorithm (v1.6.2) using default parameters. After preprocessing datasets were analyzed using the Seurat single-cell analysis pipeline (v5.0.3) in R (v4.3.1). Quality control involved excluding low-quality cells based on the following criteria: nFeature_RNA between 1000 and 7500, nCount_RNA between 2500 and 40000, and mitochondrial contamination below 10%. Following filtering data were normalized using the NormalizeData function with the "LogNormalize" method and the 2000 most variable genes were identified and scaled before performing PCA. Thirty principal components were used for graph-based clustering (resolution = 0.5) followed by UMAP dimensionality reduction.

An additional filtering step based on tdTomato expression removed contaminant clusters with fewer than 0.01% tdTomato+ cells and fewer than 5 absolute tdTomato+ cells. Initial cell type annotation was conducted automatically using scType (58) followed by manual refinement. Differential gene expression (DE) analysis between cell types in the small intestine samples were conducted using Wilcoxon rank sum tests as implemented in the Seurat function FindAllMarkers. For DE anlaysis between tissues, counts from each sample were summed and followed by pseudobulk DE analysis using limma-voom.

For manuscripts utilizing custom algorithms or software that are central to the research but not yet described in published literature, software must be made available to editors and reviewers. We strongly encourage code deposition in a community repository (e.g. GitHub). See the Nature Portfolio guidelines for submitting code & software for further information.

Data

Policy information about availability of data

All manuscripts must include a data availability statement. This statement should provide the following information, where applicable:

- Accession codes, unique identifiers, or web links for publicly available datasets
- A description of any restrictions on data availability
- For clinical datasets or third party data, please ensure that the statement adheres to our policy

All raw and processed scRNA-seq data generated in this study have been deposited in the NCBI Gene Expression Omnibus (GEO) under accession numbers GSE280635. All the other data supporting the findings of this study are available.

Research involving human participants, their data, or biological material

Policy information about studies with human participants or human data. See also policy information about sex, gender (identity/presentation), and sexual orientation and race, ethnicity and racism.

Reporting on sex and gender	Primary cell lines established from gut tissues of two male and one female human subjects.
Reporting on race, ethnicity, or other socially relevant groupings	N/A
Population characteristics	N/A
Recruitment	Patients underwent colonoscopy or sigmoidoscopy for non-inflammatory indications (e.g., colorectal cancer screening) and were referred as healthy controls (HC). Baseline demographic information for the study participants are provided in Supplementary Table S1. Patients consented to publish de-identified patient demographics including age at the time of sample collection, sex, diagnosis, and medical center. Demographic options were defined by the investigators and participants chose their classifications.
Ethics oversight	The study was conducted in accordance with the principles of the Declaration of Helsinki and was approved by the Institutional Review Board of the University of California, San Francisco (19-27302). All participants provided written informed consent before inclusion.

Note that full information on the approval of the study protocol must also be provided in the manuscript.

Field-specific reporting

Please select the one below that is the best fit for your research. If you are not sure, read the appropriate sections before making your selection.

☒ Life sciences ☐ Behavioural & social sciences ☐ Ecological, evolutionary & environmental sciences

For a reference copy of the document with all sections, see nature.com/documents/nr-reporting-summary-flat.pdf

Life sciences study design

All studies must disclose on these points even when the disclosure is negative.

Sample size	*Describe how sample size was determined, detailing any statistical methods used to predetermine sample size OR if no sample-size calculation was performed, describe how sample sizes were chosen and provide a rationale for why these sample sizes are sufficient.*
Data exclusions	*Describe any data exclusions. If no data were excluded from the analyses, state so OR if data were excluded, describe the exclusions and the rationale behind them, indicating whether exclusion criteria were pre-established.*
Replication	*Describe the measures taken to verify the reproducibility of the experimental findings. If all attempts at replication were successful, confirm this OR if there are any findings that were not replicated or cannot be reproduced, note this and describe why.*
Randomization	*Describe how samples/organisms/participants were allocated into experimental groups. If allocation was not random, describe how covariates were controlled OR if this is not relevant to your study, explain why.*

Blinding | *Describe whether the investigators were blinded to group allocation during data collection and/or analysis. If blinding was not possible, describe why OR explain why blinding was not relevant to your study.*

Reporting for specific materials, systems and methods

We require information from authors about some types of materials, experimental systems and methods used in many studies. Here, indicate whether each material, system or method listed is relevant to your study. If you are not sure if a list item applies to your research, read the appropriate section before selecting a response.

Materials & experimental systems

n/a	Involved in the study	
☐	☒	Antibodies
☐	☒	Eukaryotic cell lines
☒	☐	Palaeontology and archaeology
☐	☒	Animals and other organisms
☒	☐	Clinical data
☒	☐	Dual use research of concern
☒	☐	Plants

Methods

n/a	Involved in the study	
☐	☐	ChIP-seq
☐	☒	Flow cytometry
☐	☐	MRI-based neuroimaging

Antibodies

Antibodies used | All antibodies used have been detailed described in the manuscript.

Validation | Antibodies were purchased from reliable commercial sources.

Antibodies	Suppliers	Catalogs	Dilution	Website
Brilliant Violet 421™ anti-mouse CD19 Antibody	BioLegend	115549	1:200	https://www.biolegend.com/fr-lu/products/brilliant-violet-421-anti-mouse-cd19-antibody-7160
Brilliant Violet 421™ anti-mouse TER-119/Erythroid Cells Antibody	BioLegend	116234	1:200	https://www.biolegend.com/fr-lu/products/brilliant-violet-421-anti-mouse-ter-119-erythroid-cells-antibody-7259
Brilliant Violet 421™ anti-mouse Ly-6G/Ly-6C (Gr-1) Antibody	BioLegend	108445	1:200	https://www.biolegend.com/fr-lu/products/brilliant-violet-421-anti-mouse-ly-6g-ly-6c-gr-1-antibody-7201
Brilliant Violet 605™ anti-human/mouse/rat CD278 (ICOS) Antibody	BioLegend	313538	1:100	https://www.biolegend.com/fr-lu/products/brilliant-violet-605-anti-human-mouse-rat-cd278-icos-antibody-14371
Brilliant Violet 421™ anti-mouse/human CD11b Antibody	BioLegend	101251	1:200	https://www.biolegend.com/fr-lu/products/brilliant-violet-421-anti-mouse-human-cd11b-antibody-7163
Pacific Blue™ anti-mouse CD11c Antibody	BioLegend	117322	1:200	https://www.biolegend.com/fr-lu/products/pacific-blue-anti-mouse-cd11c-antibody-3864
Pacific Blue™ anti-mouse CD49b (pan-NK cells) Antibody	BioLegend	108918	1:200	https://www.biolegend.com/fr-lu/products/pacific-blue-anti-mouse-cd49b-pan-nk-cells-antibody-6571
Brilliant Violet 421™ anti-mouse CD335 (NKp46) Antibody	BioLegend	137612	1:200	https://www.biolegend.com/fr-lu/products/brilliant-violet-421-anti-mouse-cd335-nkp46-antibody-7506
Brilliant Violet 421™ anti-mouse NK-1.1 Antibody	BioLegend	108741	1:200	https://www.biolegend.com/fr-lu/products/brilliant-violet-421-anti-mouse-nk-1-1-antibody-7150
BrilliantViolet 421™ anti-mouse TCR γ/δ Antibody	BioLegend	118120	1:200	https://www.biolegend.com/fr-lu/products/brilliant-violet-421-anti-mouse-tcr-gamma-delta-antibody-7249
Pacific Blue™ anti-mouse FcεRIα Antibody	BioLegend	134314	1:200	https://www.biolegend.com/fr-lu/products/pacific-blue-anti-mouse-fcepsilonrialpha-antibody-6703
Pacific Blue™ anti-mouse F4/80 Antibody	BioLegend	123124	1:200	https://www.biolegend.com/fr-lu/products/pacific-blue-anti-mouse-f4-80-antibody-4075
BUV395 Rat Anti-Mouse CD45	BD	564279	1:200	https://www.bdbiosciences.com/en-us/products/reagents/flow-cytometry-reagents/research-reagents/single-color-antibodies-ruo/buv395-rat-anti-mouse-cd45.564279?tab=product_details
PE/Cyanine7 anti-mouse/human KLRG1 (MAFA) Antibody	BioLegend	138416	1:100	https://www.biolegend.com/fr-lu/products/pe-cyanine7-anti-mouse-human-klrg1-mafa-antibody-8312
APC anti-human CD4 Antibody	BioLegend	300514	1:50	https://www.biolegend.com/fr-lu/products/apc-anti-human-cd4-antibody-823
Ki-67 Monoclonal Antibody (SolA15), FITC, eBioscience™	Invitrogen	11-5698-82	1:1000	https://www.thermofisher.com/antibody/product/Ki-67-Antibody-clone-SolA15-Monoclonal/11-5698-82
APC anti-mouse CD201 (EPCR) Antibody	BioLegend	141506	1:200	https://www.biolegend.com/fr-lu/products/apc-anti-mouse-cd201-epcr-antibody-7886
PerCP/Cyanine5.5 anti-mouse CD31 Antibody	BioLegend	102420	1:200	https://www.biolegend.com/fr-lu/products/percp-cyanine5-5-anti-mouse-cd31-antibody-6668
Brilliant Violet 421™ anti-mouse CD31 Antibody	BioLegend	102424	1:200	https://www.biolegend.com/fr-lu/products/brilliant-violet-421-anti-mouse-cd31-antibody-8599
Brilliant Violet 605™ anti-mouse CD140α Antibody	BioLegend	135916	1:100	https://www.biolegend.com/fr-lu/products/brilliant-violet-605-anti-mouse-cd140a-antibody-15109

Alexa Fluor® 488 anti-mouse LYVE1 Antibody	eBiosciences	53-0443-82	1:100	https://www.thermofisher.com/antibody/product/LYVE1-Antibody-clone-ALY7-Monoclonal/53-0443-82
Alexa Fluor® 488 anti-mouse Podoplanin Antibody	BioLegend	127406	1:100	https://www.biolegend.com/fr-lu/products/alexa-fluor-488-anti-mouse-podoplanin-antibody-4751
BV786 Rat anti-mouse CD34	BD	742971	1:100	https://www.bdbiosciences.com/en-us/products/reagents/flow-cytometry-reagents/research-reagents/single-color-antibodies-ruo/bv786-rat-anti-mouse-cd34.742971?tab=product_details
Alexa Fluor® 647 anti-mouse CD326 (Ep-CAM) Antibody	BioLegend	118212	1:100	https://www.biolegend.com/fr-lu/products/alexa-fluor-647-anti-mouse-cd326-ep-cam-antibody-4973
Alexa Fluor® 488 anti-mouse CD326 (Ep-CAM) Antibody	BioLegend	118210	1:100	https://www.biolegend.com/fr-lu/products/alexa-fluor-488-anti-mouse-cd326-ep-cam-antibody-4972
Alexa Fluor® 488 Rat IgG2a, κ Isotype Ctrl Antibody	BioLegend	400525	1:100	https://www.biolegend.com/fr-lu/products/alexa-fluor-488-rat-igg2a-kappa-isotype-ctrl-2682
Anti-DCAMKL1 antibody	Abcam	ab31704	1:500	https://www.abcam.com/en-us/products/primary-antibodies/dcamkl1-antibody-ab31704?srsltid=AfmBOooWK-klGb0WmC-vZVqchamVQrtivBlWv_xQ8Y77KO2gv6dH_Bdi
Mouse Podoplanin Antibody	R&D	AF3244	1:250	https://www.rndsystems.com/products/mouse-podoplanin-antibody_af3244
Mouse PDGFR alpha Antibody	R&D	MAB1062	1:250	https://www.rndsystems.com/products/mouse-pdgf-ralpha-antibody-189208_mab1062
Mouse EPCR (CD201) Antibody	R&D	AF2749	1:200	https://www.rndsystems.com/products/mouse-epcr-antibody_af2749
CD34 Monoclonal Antibody (RAM34), eBioscience™	ThermoFisher	14-0341-82	1:500	https://www.thermofisher.com/antibody/product/CD34-Antibody-clone-RAM34-Monoclonal/14-0341-82
Anti-Green Fluorescent Protein Antibody	Aveslabs	GFP-1020	1:500	https://www.antibodiesinc.com/products/anti-green-fluorescent-protein-antibody-gfp?srsltid=AfmBOooLZXsckBI81Y2anClkRxJt3LRieoe8OCCXdFwR9xoVGy0PBNKh
Living Colors® DsRedPolyclonal Antibody	TakaraBio	632496	1:250	https://www.takarabio.com/products/antibodies-and-elisa/fluorescent-protein-antibodies/red-fluorescent-protein-antibodies?srsltid=AfmBOornk_0PBiq4yY73d8T288enog67I0VqwUNtZ4I5jSFFBzVmZx6O
mCherry Monoclonal Antibody (16D7)	ThermoFisher	M11217	1:500	https://www.thermofisher.com/antibody/product/mCherry-Antibody-clone-16D7-Monoclonal/M11217
TSLP Monoclonal Antibody	Invitrogen	MA5-23779	1:100	https://www.thermofisher.com/antibody/product/TSLP-Antibody-clone-152640-Monoclonal/MA5-23779

Eukaryotic cell lines

Policy information about cell lines and Sex and Gender in Research

Cell line source(s)	Primary intestinal fibroblasts derived from healthy donors, 2 males and 1 female; generated and provided by Michael G. Kattah
Authentication	The primary human fibroblast cell lines were originally derived by our coauthors and not commercially purchased. The methodology of deriving the cell lines have been detailed described in the Methods section. All cell lines have been grown below passage 9. The cell lines have been tested by microscopy to verify fibroblast identity and transcriptomic profiling confirming fibroblast marker gene signatures.
Mycoplasma contamination	Negative for Mycoplasma
Commonly misidentified lines (See ICLAC register)	N/A

Animals and other research organisms

Policy information about studies involving animals; ARRIVE guidelines recommended for reporting animal research, and Sex and Gender in Research

Laboratory animals	Mouse
Wild animals	N/A
Reporting on sex	Both female and male mice were used; sex-bias (if any) was reported in the manuscript.
Field-collected samples	N/A
Ethics oversight	UCSF Institutional Animal Care and Use Committee (IACUC); UCSF Laboratory Animal Resource Center (LARC)

Note that full information on the approval of the study protocol must also be provided in the manuscript.

Plants

Seed stocks | *Report on the source of all seed stocks or other plant material used. If applicable, state the seed stock centre and catalogue number. If plant specimens were collected from the field, describe the collection location, date and sampling procedures.*

Novel plant genotypes | *Describe the methods by which all novel plant genotypes were produced. This includes those generated by transgenic approaches, gene editing, chemical/radiation-based mutagenesis and hybridization. For transgenic lines, describe the transformation method, the number of independent lines analyzed and the generation upon which experiments were performed. For gene-edited lines, describe the editor used, the endogenous sequence targeted for editing, the targeting guide RNA sequence (if applicable) and how the editor was applied.*

Authentication | *Describe any authentication procedures for each seed stock used or novel genotype generated. Describe any experiments used to assess the effect of a mutation and, where applicable, how potential secondary effects (e.g. second site T-DNA insertions, mosiacism, off-target gene editing) were examined.*

ChIP-seq

Data deposition

☐ Confirm that both raw and final processed data have been deposited in a public database such as GEO.

☐ Confirm that you have deposited or provided access to graph files (e.g. BED files) for the called peaks.

Data access links
May remain private before publication. | *For "Initial submission" or "Revised version" documents, provide reviewer access links. For your "Final submission" document, provide a link to the deposited data.*

Files in database submission | *Provide a list of all files available in the database submission.*

Genome browser session
(e.g. UCSC) | *Provide a link to an anonymized genome browser session for "Initial submission" and "Revised version" documents only, to enable peer review. Write "no longer applicable" for "Final submission" documents.*

Methodology

Replicates | *Describe the experimental replicates, specifying number, type and replicate agreement.*

Sequencing depth | *Describe the sequencing depth for each experiment, providing the total number of reads, uniquely mapped reads, length of reads and whether they were paired- or single-end.*

Antibodies | *Describe the antibodies used for the ChIP-seq experiments; as applicable, provide supplier name, catalog number, clone name, and lot number.*

Peak calling parameters | *Specify the command line program and parameters used for read mapping and peak calling, including the ChIP, control and index files used.*

Data quality | *Describe the methods used to ensure data quality in full detail, including how many peaks are at FDR 5% and above 5-fold enrichment.*

Software | *Describe the software used to collect and analyze the ChIP-seq data. For custom code that has been deposited into a community repository, provide accession details.*

Flow Cytometry

Plots

Confirm that:

☐ The axis labels state the marker and fluorochrome used (e.g. CD4-FITC).

☐ The axis scales are clearly visible. Include numbers along axes only for bottom left plot of group (a 'group' is an analysis of identical markers).

☒ All plots are contour plots with outliers or pseudocolor plots.

☒ A numerical value for number of cells or percentage (with statistics) is provided.

Methodology

Sample preparation | Standard live cell surface antibody staining and intracellular staining

Instrument | BD Fortessa; BD Aria;Cytek Aurora

Software | FlowJo

Cell population abundance	Reported in the manuscript
Gating strategy	Included in the supplementary

☒ Tick this box to confirm that a figure exemplifying the gating strategy is provided in the Supplementary Information.

Magnetic resonance imaging

Experimental design

Design type	*Indicate task or resting state; event-related or block design.*
Design specifications	*Specify the number of blocks, trials or experimental units per session and/or subject, and specify the length of each trial or block (if trials are blocked) and interval between trials.*
Behavioral performance measures	*State number and/or type of variables recorded (e.g. correct button press, response time) and what statistics were used to establish that the subjects were performing the task as expected (e.g. mean, range, and/or standard deviation across subjects).*

Acquisition

Imaging type(s)	*Specify: functional, structural, diffusion, perfusion.*
Field strength	*Specify in Tesla*
Sequence & imaging parameters	*Specify the pulse sequence type (gradient echo, spin echo, etc.), imaging type (EPI, spiral, etc.), field of view, matrix size, slice thickness, orientation and TE/TR/flip angle.*
Area of acquisition	*State whether a whole brain scan was used OR define the area of acquisition, describing how the region was determined.*

Diffusion MRI ☐ Used ☐ Not used

Preprocessing

Preprocessing software	*Provide detail on software version and revision number and on specific parameters (model/functions, brain extraction, segmentation, smoothing kernel size, etc.).*
Normalization	*If data were normalized/standardized, describe the approach(es): specify linear or non-linear and define image types used for transformation OR indicate that data were not normalized and explain rationale for lack of normalization.*
Normalization template	*Describe the template used for normalization/transformation, specifying subject space or group standardized space (e.g. original Talairach, MNI305, ICBM152) OR indicate that the data were not normalized.*
Noise and artifact removal	*Describe your procedure(s) for artifact and structured noise removal, specifying motion parameters, tissue signals and physiological signals (heart rate, respiration).*
Volume censoring	*Define your software and/or method and criteria for volume censoring, and state the extent of such censoring.*

Statistical modeling & inference

Model type and settings	*Specify type (mass univariate, multivariate, RSA, predictive, etc.) and describe essential details of the model at the first and second levels (e.g. fixed, random or mixed effects; drift or auto-correlation).*
Effect(s) tested	*Define precise effect in terms of the task or stimulus conditions instead of psychological concepts and indicate whether ANOVA or factorial designs were used.*

Specify type of analysis: ☐ Whole brain ☐ ROI-based ☐ Both

Statistic type for inference (See Eklund et al. 2016)	*Specify voxel-wise or cluster-wise and report all relevant parameters for cluster-wise methods.*
Correction	*Describe the type of correction and how it is obtained for multiple comparisons (e.g. FWE, FDR, permutation or Monte Carlo).*

Models & analysis

n/a	Involved in the study
☐	☐ Functional and/or effective connectivity
☐	☐ Graph analysis
☐	☐ Multivariate modeling or predictive analysis

Functional and/or effective connectivity	*Report the measures of dependence used and the model details (e.g. Pearson correlation, partial correlation, mutual information).*
Graph analysis	*Report the dependent variable and connectivity measure, specifying weighted graph or binarized graph, subject- or group-level, and the global and/or node summaries used (e.g. clustering coefficient, efficiency, etc.).*
Multivariate modeling and predictive analysis	*Specify independent variables, features extraction and dimension reduction, model, training and evaluation metrics.*

This checklist template is licensed under a Creative Commons Attribution 4.0 International License, which permits use, sharing, adaptation, distribution and reproduction in any medium or format, as long as you give appropriate credit to the original author(s) and the source, provide a link to the Creative Commons license, and indicate if changes were made. The images or other third party material in this article are included in the article's Creative Commons license, unless indicated otherwise in a credit line to the material. If material is not included in the article's Creative Commons license and your intended use is not permitted by statutory regulation or exceeds the permitted use, you will need to obtain permission directly from the copyright holder. To view a copy of this license, visit http://creativecommons.org/licenses/by/4.0/
